# Re-introduction of India ink testing as a low-cost laboratory diagnostic for cryptococcosis among HIV infected patients in Southern Mozambique: An implementation research protocol

**DOI:** 10.1371/journal.pone.0324792

**Published:** 2025-05-23

**Authors:** José C. Langa, Mohsin Sidat, Jahit Sacarlal, Troy D. Moon

**Affiliations:** 1 Department of Microbiology, Faculty of Medicine, Eduardo Mondlane University, Maputo, Mozambique; 2 Department of Community Health, Faculty of Medicine, Eduardo Mondlane University, Maputo, Mozambique; 3 Department of Tropical Medicine and Infectious Diseases, Tulane University School of Public Health and Tropical Medicine, New Orleans, Louisiana, United States of America; Gulu University, UGANDA

## Abstract

Laboratory diagnosis for cryptococcal disease among HIV-infected patients remains a challenge in most low- and middle-income countries (LMIC). Difficulties with sustained access to cryptococcal rapid tests is cited as a major barrier to the routine screening for cryptococcus in many LMIC. Thus, clinicians in these countries often resort to empirical treatment based solely on clinical suspicion of cryptococcosis. To address this challenge, we aim to evaluate the re-introduction of India ink testing for diagnosis of cryptococcosis among HIV-infected patients in southern Mozambique. India ink testing was historically a common first choice, low-cost, laboratory diagnostic tool for cryptococcal infection. This study uses implementation science methods framed by the Dynamic Adaption Process (DAP) and the Reach, Effectiveness, Adoption, Implementation, and Maintenance (RE-AIM) conceptual frameworks to develop a multi-phase, stepped-wedged trial using mixed-methods approaches. The study will be conducted in six hospitals from southern Mozambique over a period of 15 months and will include the following phases: pre-implementation (baseline assessment), Adaptation-implementation (gradual introduction of the intervention), and post-implementation (post-intervention assessment). This study aims to promote the use of India Ink staining as a cheap and readily available tool for cryptococcosis diagnosis in southern Mozambique. Lessons learned in this study may be important to inform approaches to overcome the existing challenges in diagnosis of cryptococcosis in many LMICs due unavailability of readily diagnostic tools. **Trial registration:** ISRCTN11882960, Registered 06 August 2024.

## Introduction

Worldwide, cryptococcal infections have increased due to the HIV/AIDS pandemic, particularly in sub-Saharan Africa (SSA) where the associated mortality is relatively high [1,2]. In 2020, the global annual incidence of cryptococcosis was estimated at 152,000 cases, resulting in roughly 112,000 cryptococcosis-related deaths [3]. More than 70% of cryptococcosis deaths occur in low- and middle-income countries (LMIC) [2,3].

HIV-associated cryptococcosis is a major cause of hospital admissions and clinic visits in LMIC [3,4], particularly affecting the most economically productive age groups. This is especially true in SSA where opportunistic infections such as cryptococcosis continues to be a common occurrence among HIV-infected patients, despite recent efforts to expand access to antiretroviral therapy (ART) [2,5,6]. Nevertheless, as much as 75% of HIV-infected adults in LMIC do not have access to cryptococcal diagnostic testing, and suspected cases are commonly treated empirically [2,4].

In Mozambique, the prevalence of HIV-infection in the adult population is currently estimated at 12.5% [7]. Accurate data on cryptococcosis prevalence in the country are scarce and most often patients are diagnosed based only on clinical suspicion. It is estimated that the number of cryptococcosis cases in the country are around 18,600 cases per year (70.5 cases per 100,000 persons) [2,8], and in one study of hospitalized HIV patients in southern Mozambique, the prevalence was approximately 5.3% [9].

Health care facilities in resource-limited settings frequently experience a variety of different challenges such as financial constraints, scarcity of laboratory consumables, lack of basic essential equipment, limited numbers of skilled personnel, lack of educators and training programs, and inadequate logistical support. Consequently, limited or no access to point-of-care (POC) diagnostics negatively impacts care and results in both delays in diagnosis and delays in initiating treatments [4,6]. These limitations likely contribute to 1) patient loss-to-follow-up due to a need for frequent visits to the facility to get results; 2) diagnosis being made empirically, based solely on clinical symptoms which can be nonspecific, unreliable, and may contribute to an increase in mortality; and/or 3) no diagnosis being made at all, due to a lack of awareness or due to limited clinician training with regards to clinically complex cases [10–12].

India ink microscopy has been used to diagnose cryptococcosis for more than a century [13]. This method is relatively simple, inexpensive, and easy to perform in the laboratory, however, the introduction and expansion of rapid diagnostic tests (RDT) for a variety of illnesses has contributed to an overall reduction in the use of microscopy [13,14]. Furthermore, India ink microscopy specifically, had fallen out of favor as a diagnostic test due to it being associated with a relatively low sensitivity (approximately 70–90%) and due to the introduction of newer RDT´s that were anticipated to be a superior alternative to India ink [13–15].

The rapid cryptococcal antigen (CrAg) test, for example, has shown to have good sensitivity and specificity for diagnosing cryptococcosis. However, in some resource-limited settings it has not reached its full potential and its utilization for routine cryptococcal diagnosis remains limited [14,15].

In Mozambique, for example, the National Health System (NHS) has had challenges maintaining appropriate stocks of the CrAg RDT and has faced difficulties in ensuring quality control of the tests that are performed [13]. As such, cryptococcosis in Mozambique is significantly underdiagnosed, or is empirically diagnosed only once a patient´s symptoms have progressed to being severe and in advanced stages.

To address this, we propose an initiative to reintroduce the routine use of India ink microscopy on urine samples as an alternative to CrAg for cryptococcosis diagnosis, particularly among HIV-infected patients. Studies have shown that in persons infected with HIV, cryptococcus can be eliminated in the urine on average 22 days before the onset of cryptococcal meningitis (CM) symptoms [16,17]. Even with its lower reported sensitivity as a screening test, in the absence of good access to CrAg or other definitive diagnostics, we feel that routine testing of urine with India ink constitutes a “window” of opportunity for screening and subsequent initiation of treatment prior to the full establishment of cryptococcal meningitis manifestations. In this context, urine samples can be easily collected and tested for cryptococcosis at a relatively early stage of systemic infection, without need for invasive procedures such as lumbar puncture.

Therefore, we hypothesize that the adoption of India ink testing as a low-cost laboratory diagnostic for cryptococcosis in the hospitals will increase the opportunities to diagnose the disease among HIV- infected patients during the intervention period as compared to the control period.

## Materials and methods

This protocol was developed following the SPIRIT guidelines (Standard Protocol Items: Recommendations for Intervention Trials)[18].

### Study aim

The overall aim of this study is to evaluate the re-introduction of India ink microscopy of urine samples, as a low-cost, POC diagnostic test for HIV-infected patients suspected of having cryptococcal disease.

### Objectives

1) To explore the opportunities and barriers to re-introduction of India ink microscopy of urine samples, as a diagnostic test for cryptococcal infections among HIV-infected patients in southern Mozambique (Pre-implementation phase).
2) To develop strategies for implementing low-cost, POC diagnostic test for cryptococcal infection in southern Mozambique (Adaptation and implementation phase)
3) To assess the reach, effectiveness, adoption, implementation, and maintenance of the intervention in Southern Mozambique (Post-implementation phase).

### Expected results of the research

1) Make available a low-cost POC laboratory-backed diagnosis of cryptococcal infection for HIV-infected patients in study sites and eventually expand nationwide.
2) Train laboratory technicians to perform microscopy to diagnose cryptococcal infection and interpret results.
3) Raise awareness on the occurrence of cryptococcal infection among HIV-infected patients in the country and the possibility of performing an etiologic diagnosis of suspected cases by using a low-cost POC technique (which we expect to introduce nationwide)

### Study setting

**Fig 1:**
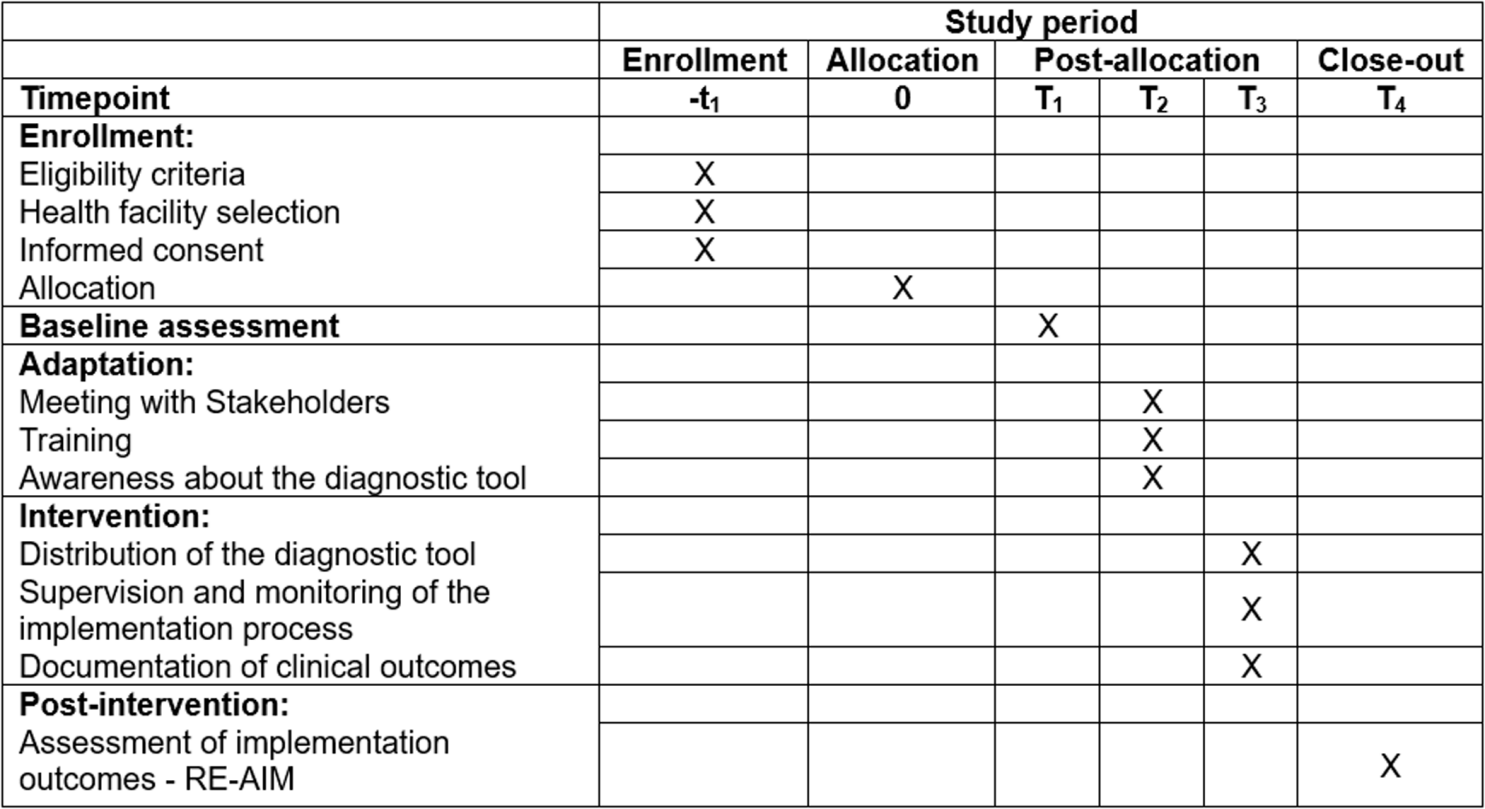
The SPIRIT schedule of enrollment, intervention, and assessments.

**Fig 2:**
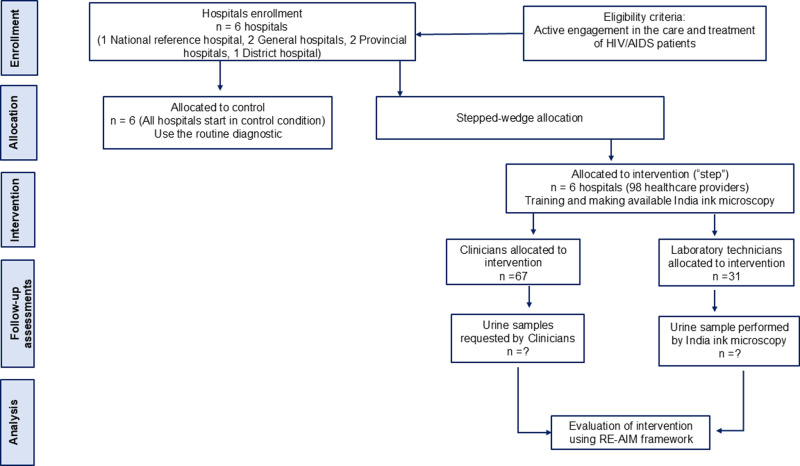
The SPIRIT flow diagram.

This study will take place in six hospitals conveniently selected within Maputo and Gaza Provinces, in the southern part of Mozambique. The study sites are public hospitals, part of the National Health Services (NHS), that were selected because of their active engagement in the care and treatment of HIV/AIDS patients as well as due to the availability of microscopy testing capacity in their clinical laboratories. In Maputo Province the following hospitals were selected: Maputo Central Hospital (MCH), Mavalane General Hospital (MGH), Jose Macamo General Hospital (JMGH), and Matola Provincial Hospital (MPH). In Gaza Province the following hospitals were selected: Xai-Xai Provincial Hospital (XPH) and Carmelo Hospital of Chókwè(CHC). The SPIRIT schedule and overall study process are shown in Figs 1 and 2.

### Study design and conceptual framework

**Fig 3:**
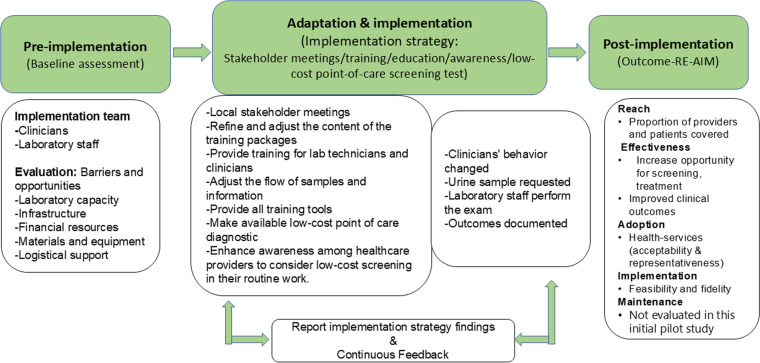
The Dynamic Adaptation Process and RE-AIM conceptual frameworks contextualized to this project.

A mixed-methods study design will be utilized and guided by two conceptual frameworks, the Dynamic Adaptation Process (DAP) and the Reach, Effectiveness, Adoption, Implementation, and Maintenance (RE-AIM). The study will be carried out in three phases (pre-implementation, adaptation and implementation, and post-implementation) (Fig 3**).** The DAP framework was developed to provide the structure for an iterative process to guide, monitor, and evaluate the introduction of a new intervention into practice [19,20]. The RE-AIM framework was developed to evaluate the intervention in terms of five domains (*Reach, Effectiveness, Adoption, Implementation, and Maintenance*) considered most relevant for real world intervention and to provide individual and organizational recommendations to enhance the healthcare program [21,22].

#### Pre-implementation phase.

During the pre-implementation phase (duration three months), we will first explore perceived barriers and opportunities, from clinicians and laboratory technicians, related to the roll-out of India Ink diagnosis of cryptococcosis on urine samples. This will be accomplished utilizing a semi-structured self-administered questionnaire as well as through in-depth interviews with clinicians and laboratory technicians at our study hospitals. In addition, we will conduct a baseline assessment of the implementation capacity of our study hospitals, exploring their overall laboratory capacity and available infrastructure, their consistency in maintaining laboratory stocks of materials and supplies to conduct microscopy, and their financial resources and other available logistic supports available.

#### Adaptation and implementation phase.

During the adaptation and implementation phase (duration nine months), initially will be conducted based on data gathered during the pre-implementation phase, the study team will conduct local stakeholder meetings with study facility staff to refine and adjust the content of the training packages offered as well as to refine and adjust the facility specific operational procedures for the implementation of India Ink microscopy, including coordination approaches between laboratory technicians and the HIV care clinicians. Subsequently, the study team will initiate training with the HIV care clinicians of our study hospitals related to the diagnosis and treatment of cryptococcosis, emphasizing diagnostic options such as India Ink microscopy on urine and interpretation of results. Additionally, training will take place with the laboratory technicians of our study hospitals on the conduct of India Ink microscopy procedures in the laboratory.

Once implementation begins at a given study site, continuous monitoring of intervention roll-out will occur such that ongoing adaptations can be made in real-time, as needed.

Finally, during the *Post-Implementation Phase* (duration three months) we will assess the intervention’s implementation at two levels: individual (healthcare providers) and organizational (hospitals and their healthcare system), using a modified RE-AIM framework (Fig 3). This study is being implemented as a pilot study with limitations in the length of time we can perform follow-up. As such, the maintenance domain of the RE-AIM framework will not be evaluated.

The RE-AIM domains by measure and data source to analyze implementation outcomes are shown in Table 1.

**Table 1:** RE-AIM domains by measure and data source.

RE-AIM dimension	RE-AIM- definition/ Description	Measure	Data source
Reach	Scope of the intervention, including healthcare providers who benefited from the intervention, in each site.	- Number of healthcare providers (clinicians and laboratory technicians) who attended the training;) - Number of services per site that have signed up to cryptococcosis diagnosis using urine; - Number of clinicians requesting a urine sample during cryptococcosis screening;	- Review the clinical processes - Review the laboratory database. - short quantitative surveys (healthcare providers)
Effectiveness	Outcomes (positive and negative) resulting from training, awareness, and the implementation of the India ink platform as a point-of-care for cryptococcosis screening.	- Change in clinician’s behavior. In their routine they chose to make a etiological laboratory diagnosis instead of empirical treatment (abandonment of empirical treatment). - Number of times the clinician supported by the new diagnosis to assist their patients. - Clinician satisfaction (responses ranged from strongly disagree (1) to strongly agree (4). Type of tests requested by clinicians to diagnose cryptococcosis. - Number of urine specimens tested by the laboratory for cryptococcal disease. - Average laboratory response time	- Review the clinical processes - Review the laboratory database. - Short quantitative surveys (healthcare providers) - Focal points (supervisors) checklist
Adoption	The department or service in each hospital that has supported the intervention (outcomes represent the acceptability and representativeness of the intervention).	Number of departments or services that: - Accepted and supported the India Ink platform using urine as a point-of-care for cryptococcal diagnosis in their services; - Supported the adjustment of sample and information flow for cryptococcosis diagnosis; - supported the introduction of guidelines for cryptococcosis diagnosis.	- Review the clinical processes - Review the laboratory database. - Short quantitative surveys (healthcare providers) - Focal points (supervisors) checklist
Implementation	Fidelity of healthcare providers in terms of the judgement, tools and resources used to manage cryptococcosis in their daily routine.	Number of healthcare providers (clinicians and laboratory technicians) who attended the training, and during their routine work assisted patients suspected of having cryptococcosis based on the new diagnostic platform.	- Review the training tools - Focal points (supervisors) checklist

### Stepped-wedge trial approach and characteristics of study participants

Our intervention, the implementation of India ink microscopy on urine samples, will be rolled out using a stepped-wedge trial approach in six hospitals conveniently selected for this study. All six hospitals will be classified at start as being in the control condition (unexposed to the intervention), as currently none of them are implementing India Ink testing for cryptococcosis. Following the pre-implementation phase, we will roll-out the intervention sequentially by including a new facility each month until all six hospitals are covered (Table 2). The stepped-wedge design was chosen because of its inherent advantages for evaluating health interventions in low-resource settings [23–25]. These advantages include greater flexibility in addressing logistical and infrastructural constraints, allowing for adaptations to be made based on initial roll-out experiences, as well as a gradual implementation that allows for: 1) better resource management, 2) health facilities that can serve as their own controls, and 3) ensuring that all facilities will eventually receive the intervention.

**Table 2:** Stepped-wedge design.

Sites					Time										
	15 months														
MCH	C	PI	AD-I	FU	FU	FU	FU	FU	FU	FU	FU	FU	FU	FU	FU
MGH	C	C	PI	AD-I	FU	FU	FU	FU	FU	FU	FU	FU	FU	FU	FU
JMGH	C	C	C	PI	AD-I	FU	FU	FU	FU	FU	FU	FU	FU	FU	FU
MPH	C	C	C	C	PI	AD-I	FU	FU	FU	FU	FU	FU	FU	FU	FU
XPH	C	C	C	C	C	PI	AD-I	FU	FU	FU	FU	FU	FU	FU	FU
CHC	C	C	C	C	C	C	PI	AD-I	FU	FU	FU	FU	FU	FU	FU

Note: C- Control; PI- Pre-implementation (baseline); AD-I- Adaptation & implementation; FU- Follow-up (Evaluation process)

### Study participants

The study will include two types of participants, namely: clinicians engaged in HIV/AIDS care and treatment; and laboratory technicians who have been tasked with conducting laboratory diagnostic testing for cryptococcosis. During the pre-implementation phase, the eligible participants from across our six study facilities (67 clinicians and 31 laboratory technicians) will be informed about the study and how it will be carried out during all three phases in their health facilities. The research team will be responsible for obtaining the written informed consent from each participant enrolled in the study. Once enrolled, all healthcare professionals will be involved in all three phases of the study.

Will be excluded from the study the clinicians attending to pediatric patients; clinicians who are not engaged in HIV/AIDS care and treatment and the laboratory technicians who are not engaged in laboratory diagnostic testing for cryptococcosis.

### Trial status

Recruitment of participants commenced on December 01, 2024, and will end on May 31, 2025.

### Intervention

Following completion of the pre-implementation phase assessments and local stakeholder adaptation meetings, each study facility will roll-out India Ink microscopy for cryptococcosis diagnosis as per the stepped-wedge design. Upon initiation of implementation, HIV care clinicians allocated to intervention will identify suspected cryptococcosis patients based on clinical suspicion. Urine will be requested and submitted to the laboratory on the same day for microscopy testing using India Ink. Once at the laboratory, the urine sample will be analyzed by laboratory technicians trained in the diagnosis of cryptococcosis by the study (Fig 2). In order to standardize procedures across all laboratories of the study, the laboratory technicians will follow the same Standard Operating Procedure (SOP) for conducting the India Ink microscopy technique on urine samples that was designed specifically for this study.

Microscopy results will be available on the same day and then transferred from the laboratory back to the clinician through the sample and information flow system that has been adapted for this study to enhance the interaction between clinicians and laboratory staff.

### Data collection

We will collect quantitative data from direct observation and self-administered questionnaires. Direct observation will be used to assess the availability of the material and equipment for cryptococcoses diagnosis. Questionaries will be used to assess practices and clinical conduct regarding cryptococcoses diagnosis. The questionnaires will include close-ended questions designed to capture socio demographic information and the clinical approaches to cryptococcoses diagnosis.

Qualitative data will be collected through in-depth interviews to explore barriers and facilitators for the implementation of India ink microscopy on urine samples.

Interviews will be conducted with identified clinicians and laboratory technicians in a quiet, private location and will be audio recorded. Data saturation will be met at the point at which no new information or themes are observed [26].

Data to assess the adherence of healthcare providers to our intervention will be collected from the laboratory record book. The information to be collected includes urine requested for cryptococcosis diagnosis and who requested it.

### Data management

We will design our data collection forms using a Research Electronic Data Capture (REDCap) database, a secure web-based platform to collect, store, and analyze basic data from the desired population. Quantitative data obtained through direct observation and from self-administered questionnaires will be linked to REDCap and analyzed using the R studio statistical package.

The audio recordings will be transcribed word-for-word and subsequently coded. We will carry out quality control by comparing what has been transcribed with the audio recording to ensure that no information has been omitted or added. We will categorize and organize the data as an initial thematic analysis, allowing us to identify recurrent themes.

Quantitative and qualitative data will be stored and identified in REDCap with access to all study researchers and the REDCap Data Manager of the Faculty of Medicine of Eduardo Mondlane University.

### Statistical analysis

#### Quantitative data analysis.

First, we will perform the descriptive statistics (demographic characteristics) of the participants such as mean or median age, and we will calculate the proportion of the following variables (human resources available in each site, gender, years of practice, physicians’ category, type of test requested/performed to manage cryptococcal disease and microscopes available). We will also determine the reach of the training section (the proportion of clinicians and laboratory staff who benefited from training during the adaptation and implementation phase in each hospital. Second, we will use the chi-squared test to compare years in practice and category with the type of test requested. Third, the correlation will be used to assess the association between the variables: number of participants trained and the number of cryptococcal urine tests requested/performed during the intervention phase. Fourth, to explore 1) the association between the clinician’s level of education or physicians’ category and treatment options (empirical or laboratory-based), and 2) the association between the patient’s clinical factors and the clinician’s decision related to the diagnosis and cryptococcoses treatment we will perform multivariate logistic regression.

We will use fixed effects models to analyse the overall effect of our strategy on the re-introduction of India ink testing as a laboratory diagnostic for cryptococcosis among HIV-infected patients[27]. To adjust for clustering and temporal trends, we will include the clusters (hospitals) as a categorical predictor and the time in months as a fixed effect in the model. To evaluate the opportunities for diagnosing and treating cryptococcosis among HIV-infected patients between the control condition and intervention periods, we will compare the outcomes of hospitals in the intervention condition with the outcomes of hospitals in the control condition within the same time period. We will also do the comparisons in the same hospital [28,29].

A p-value <0.05 will be considered significant level. Quantitative data analysis will be performed with the statistical software R version 4.4.1.

#### Qualitative analysis.

The participant’s information will be anonymized using the numbers and initials of their names. The qualitative data will be coded, and to analyze them, we will use Nvivo version 12, the qualitative software. All the audio recordings from the participants will be transcribed and coded using constructs compatible with the study objectives and implementation outcomes.

Triangulation between qualitative and quantitative data will be explored to identify areas of convergent and divergent responses.

### Ethical considerations

The study has been reviewed and approved by the National Bioethics Committee for Health of Mozambique (Comité Nacional de Bioética para Saúde) 48/CNBS/23) and has also received administrative approval from the following hospitals: MCH, MGH, JMGH, MPH, XPH, and CHC. All healthcare providers will write an informed consent form before answering the questionnaire and attending the interview. All participants have the right to withdraw from the study at any time.

However, the re-introduction of India ink microscopy on urine samples as an alternative diagnostic test for cryptococcosis in HIV-infected patients are deemed a negligible risk to the participants (clinicians and laboratory technicians) who adopted the intervention, even for the patients who will be reached by the intervention, and therefore no compensation plan was put in place.

## Discussion

Cryptococcosis is a life-threatening opportunistic fungal disease [30]. Early detection and management of cryptococcal infection among HIV-infected patients can prevent complications from disease and reduce mortality [6]. However, many challenges remain in the diagnosis and management of cryptococcosis in sub-Saharan Africa [1,13]. International protocols for cryptococcal diagnosis among HIV-infected patients have promoted utilization of the CrAg diagnostic test due to its favorable sensitivity and specificity and its potential as a rapid POC diagnostic test. However, the reality in Mozambique has been that CrAG is not readily available, and the relatively simple microscopy technique has become a lost skill among most laboratory technicians. As a result, cryptococcal management has been based on clinical suspicion and subsequent empirical treatment.

We propose the reintroduction of the India Ink microscopy technique on urine samples in patients suspected of having cryptococcosis. The stepped-wedge design of our intervention roll-out will strengthen our results, through providing a scenario through which each site will serve as its own control and through which the study team can manage the limited resources for intervention roll-out. Additionally, by framing our work within the context of the DAP conceptual framework, roll-out of India Ink microscopy will be informed by stakeholder perceptions of barriers and opportunities and a baseline assessment of current facility capacity. The DAP framework then allows for continuous feedback and ability to implement adaptations throughout intervention implementation.

Overall, we expect that the findings from this study could be scaled to other health facilities in Mozambique. Furthermore, the findings of this study may be useful for other countries with resource-limited socio-economic and health system contexts such as Mozambique.

### Limitations

The limitations of our study include the selection of physicians and specialists that more frequently engage in attending adult HIV-infected patients. In addition, our intervention requires expertise in microscopy management to provide the correct diagnosis and enhance clinical decision-making.

Potential bias could result from non-response to specific questions, once, the participants can answer to please their health facility and its managers.

### Dissemination plans

The findings of this study will be discussed with all healthcare providers of our study sites, the scientific community, and the Ministry of Health. The findings will also be presented at scientific conferences and published in peer-reviewed international journals.

## Supporting information

S1 File Approved protocol by ethics committee, Portuguese version. (DOCX)

S2 File SPIRIT fillable checklist. (PDF)

S3 File Translation approved protocol, English version. (DOCX)

## Abbreviations

ART: Antiretroviral therapy
CHC: Carmelo Hospital of Chókwè
CM: Cryptococcal meningitis
DAP: Dynamic Adaptation Process
JMGH: Jose Macamo General Hospital
LMIC: Low- and middle-income countries
MCH: Maputo Central Hospital
MGH: Mavalane General Hospital
MPH: Matola Provincial Hospital
PLHIV: People living with HIV
RE-AIM: Reach, Effectiveness, Adoption, Implementation, and Maintenance
XPH: Xai-Xai Provincial Hospital
