# Supplementary material for: Re-introduction of India ink testing as a low-cost laboratory diagnostic for cryptococcosis among HIV infected patients in Southern Mozambique: An implementation research protocol
Source: PLoS One. 2025 May 23;20(5):e0324792. doi: 10.1371/journal.pone.0324792 (PMC12101839; doi:10.1371/journal.pone.0324792)
Supplement: S1 File — (DOCX) [file pone.0324792.s001.docx]

**DOUTORAMENTO EM BIOCIÊNCIAS E SAÚDE PÚBLICA**

Título do Projecto de Investigação

**Implementação da técnica de tinta da China em urina para triagem laboratorial *point-of-care* da Criptococose em pacientes adultos infectados por HIV no sul de Moçambique**

Versão 04, Outubro 2023

Maputo, 11/2023

**DOUTORAMENTO EM BIOCIÊNCIAS E SAÚDE PÚBLICA**

Título do Projecto de Investigação

**Implementação da técnica de tinta da China em urina para triagem laboratorial *point-of-care* da Criptococose em pacientes adultos infectados por HIV no sul de Moçambique**

Nome do estudante: José Carlos Langa, Faculdade de Medicina, UEM

Supervisores:

Supervisor Principal: Professor Mohsin Sidat, Faculdade de Medicina, UEM

Co-Supervisor: Professor Jahit Sacarlal, Faculdade de Medicina UEM

Co-Supervisor: Professor Troy D. Moon, Tulane University School of Public Health and Tropical Medicine-USA

Versão 04, Outubro 2023

Maputo, 11/2023

**Índice**

[Resumo 6](#_Toc132207739)

[1. Motivação 7](#_Toc132207740)

[2. Objectivos 8](#_Toc132207741)

[3. Contribuição 9](#_Toc132207742)

[4. Problema 9](#_Toc132207743)

[4.1 Questões de pesquisa 10](#_Toc132207744)

[5. Revisão bibliográfica 10](#_Toc132207747)

[6. Enquadramento conceptual 13](#_Toc132207748)

[7. Metodologia 16](#_Toc132207749)

[7.1 Desenho e tipo de estudo 16](#_Toc132207750)

[7.2 Local do estudo 17](#_Toc132207751)

[7.3 Período do estudo 18](#_Toc132207752)

[7.4 População do estudo 18](#_Toc132207753)

[7.5 Amostra e amostragem 18](#_Toc132207754)

[7.6. Critério de inclusão e exclusão 19](#_Toc132207755)

[7.8. Estudos 21](#_Toc132207756)

[7.9. Variáveis e Plano de análise de dados 25](#_Toc132207759)

[8. Limitações do estudo 27](#_Toc132207760)

[9. Considerações éticas 27](#_Toc132207761)

[9.1. Potenciais riscos e como estes serão minimizados 28](#_Toc132207762)

[9.2. Consentimento informado 28](#_Toc132207763)

[9.3. Confidencialidade 28](#_Toc132207764)

[9.4. Potenciais benefícios 29](#_Toc132207765)

[9.5. Declaração de conflitos de interesse 29](#_Toc132207766)

[9.6. Resultados esperados 30](#_Toc132207768)

[9.7. Disseminação de resultados 30](#_Toc132207769)

[10. Cronograma do estudo 31](#_Toc132207770)

[11. Equipa do estudo 32](#_Toc132207771)

[11.1. Papel de cada integrante da equipe 32](#_Toc132207772)

[12. Orçamento 33](#_Toc132207773)

[13. Referências Bibliográficas 34](#_Toc132207774)

[14. Apêndices e Anexos 37](#_Toc132207775)

**Abreviaturas e acrónimos**

ADC- Autópsia Diagnóstica Completa

CDC- Centro de Controlo e Prevenção de Doenças

CNBS- Comité de Nacional de Bioética para Saude

CrAg- Antígeno criptocócico

EUA- Estados Unidos da América

HCC-Hospital Carmelo de Chokwé

HCM- Hospital Central de Maputo

HGJM- Hospital Geral José Macamo

HGM- Hospital Geral de Mavalane

HIV-Virus de Inunodificiência Humana

ID-Identificação do doente

IFI- Infencções Fúngicas Invasivas

LCR- Liquido cefaloraquidiano

MC-Meningite Criptocócica

MISAU-Ministério da SaúdeOMS-Organização Mundial de Saúde

OR-“Odds Ratio”

PAD-Processo de Adaptação Dinâmica

PS-Profissional de saúde

SIDA-Síndrome da Imunodeficiência Adquirida

SNC-Sistema Nervoso Central

SNS-Sistema Nacional da Saúde

TARV- Tratamento anti-retroviral

TDR- Teste de diagnóstico rápido

UEM-Universidade Eduardo Mondlane

US-Unidade Sanitária

VD-Variável dependente

# Resumo

No mundo a meningite criptocócica (MC) associada à infecção por HIV tem sido apontada como sendo a causa responsável de cerca de 150.000 – 200.000 mortes por ano, ocorrendo principalmente na região da África Subsaariana, com registo acima de 70%; 135.900 [95% CI 93.900–163.900]. As limitações no diagnóstico e tratamento de pacientes com MC associada ao HIV, podem ser as prováveis causas da elevada taxa de mortalidade na região da África Subsaariana.

A Organização Mundial de Saúde (OMS) recomenda triagem e tratamento da criptococose em todos pacientes HIV para evitar complicações e reduzir a taxa de mortalidade, porém, em países com recursos limitados, há necessidade de implementar diagnóstico adequado com a realidade do pais, simples e não dispendioso de modo a evitar perca de oportunidade de diagnóstico devido a roturas dos testes rápidos. A técnica da tinta da China em urina tem sido indicada como sendo uma alternativa para o diagnóstico de *Cryptococcus*, pois através da urina facilmente pode-se detectar a presença deste agente patogénico*,* principalmente em indivíduos com infecção disseminada.

O objectivo principal do estudo é a implementação da técnica de tinta da China em urina para triagem laboratorial *point-of-care* da Criptococose em pacientes adultos infectados por HIV no sul de Moçambique.

Será realizado um estudo *stepped wedge trial* de implementação científica com abordagem mista. Amostragem será não probabilística por conveniência e intencional. A recolha de dados será por questionário, observação e entrevista semi-estruturada aos profissionais de saúde (clínicos e técnicos de laboratório). A implementação será em fases e será usado um quadro conceptual *Processo de Adaptação Dinâmica* (PAD) e depois será avaliada a intervenção usando o quadro conceptual de RE-AIM (*Reach, Effectiveness, Adoption, Implementation, and Maintenance)*.

Espera-se que com os achados deste estudo fazer advocacia de modo que o Ministério da Saúde (MISAU) possa priorizar estratégias de diagnóstico com amostras não invasivas de baixo custo e maximizar o diagnóstico, controlo e prevenção de MC que juntamente com o HIV, estão devastando a saúde e os recursos socio-económicos no País.

# Motivação

Durante a coordenação da vigilância das infecções fúngicas invasivas (IFI) no Hospital Central de Maputo verificou-se desafios relacionados ao diagnóstico e ruptura dos testes rápidos actualmente em uso o que constitui limitação para o diagnóstico atempado da criptococose. Alem disso o diagnóstico a partir da amostra invasiva LCR tem constituído uma barreira em locais com poucos profissionais de saúde com habilidade para a colheita da amostra. A técnica da tinta da China em urina é simples e menos dispendioso, tem sido indicada como sendo uma alternativa para o diagnóstico de *Cryptococcus.*

As IFI assumem ultimamente papel de destaque entre as doenças que afectam a saúde e o bem-estar da população em todo mundo. Com o advento de HIV/SIDA as IFI representam umas das principais causas de consulta, internamento e morte, principalmente na África Subsaariana. Estudos mostram que tratamentos prologados com antibacterianos e sem evolução para cura tem provável causa os fungos. A mortalidade por criptococose é praticamente inevitável na ausência de um diagnóstico e tratamento adequado. Lacunas no cuidado e diagnóstico podem contribuir para uma má gestão de infecções fúngicas, sobretudo em Moçambique onde são negligenciadas.

A criptococose aumenta a custa do HIV, segundo dados de INSIDA 2021 a prevalência de HIV na população adulta em Moçambique é de 12,5%(1), o HIV constitui um factor de risco para as IFIs, e em Moçambique o diagnóstico mais complexo de IFI é realizado nos Hospitais Centrais e laboratórios de referência académico do Departamento de Microbiologia da Faculdade de Medicina da Universidade Eduardo Mondlane (UEM). Surge como prioridade implementar um diagnóstico de criptococose adequado com a realidade do pais, simples e não dispendioso de modo a evitar perca de oportunidade de diagnóstico devido a rupturas dos testes rápidos e melhorar a gestão de IFIs no âmbito de cuidados e tratamento, que pouco tem sido feito.

A introdução do diagnóstico oportuno não invasivo, promoção do mesmo, e sua expansão com vista a abranger maior parte de pacientes HIV suspeitos de criptococose pode contribuir para um início do tratamento mais próximo do momento da admissão, melhorar o desfecho dos pacientes e contribuir para a redução da mortalidade.

# Objectivos

2.1. **Objectivo geral**

- Avaliar a implementação da técnica de tinta da China em urina como técnica de triagem laboratorial *point-of-care* da criptococose em pacientes adultos infectados por HIV no sul de Moçambique

**2.2. Objetivos específicos**

- Analisar as práticas e os desafios existentes no diagnóstico laboratorial da criptococose nos hospitais HCM, HGM, HGJM, HPM, HPX e HCC;
- Implementar a técnica de diagnóstico laboratorial da Criptococose pelo método de tinta de China em urina como *point of care* nos hospitais selecionados;
- Avaliar o alcance, eficácia, adopção, implementação e manutenção do método de tinta de China em urina como diagnóstico laboratorial *point of care* da criptococose.

# Contribuição

Providenciar cuidados de saúde aceitável na África Subsaariana é um processo complexo, devido a escassez de recursos humanos e material e o acesso a testes de diagnóstico é extremamente limitado.

A cada ano, na África Subsaariana, aproximadamente 12 milhões de pessoas morrem (2,3) e na maioria, as causas da morte não são investigadas. As poucas mortes investigadas são geralmente atribuídas a doenças infecciosas (4), mais comumente infecção por HIV, malária e tuberculose.

Na ausência de confirmação laboratorial, a precisão dessas estimativas permanece incerta.

Em Moçambique, devido ao crescente número de pessoas em risco para criptococose aliado a vários factores incluindo o HIV, a introdução do diagnóstico de criptococose oportuno, menos dispendioso com amostra não invasiva (urina) e resultado no mesmo dia da consulta, pode contribuir para o aumento da confirmação laboratorial dos casos suspeitos de criptococose e orientar os clínicos com um diagnóstico etiológico para iniciar o tratamento. Poderá ser expandido para os hospitais periféricos do país, contribuir para o diagnóstico e tratamento em pacientes onde amostras invasivas não são acessíveis, permitir iniciar o tratamento no momento mais próximo da admissão do paciente.

Pretende-se igualmente que a implementação seja sustentável, usada para o rastreio contínuo (rotina) e que *alivie* os recursos socioeconômicos no País.

# Problema

A África Subsaariana tem o maior peso de infecções invasivas/oportunistas associadas à HIV/SIDA (5). A criptococose é uma importante infecção oportunista e uma das principais causas de consulta e internamento em adultos vivendo com HIV na África Subsaariana (6).

Em Moçambique aproximadamente 2.097.000 (dois milhões e noventa e sete mil) adultos vivem com HIV, com prevalência de 12,5%, segundo dados de INSIDA 2021(1).

O diagnóstico clássico de criptococose é realizado através da pesquisa do microrganismo no líquido cefalorraquidiano (LCR) ou sangue. Actualmente as amostras invasivas são substituídas por amostras alternativas não invasivas, fáceis de obter que tem mostrado o mesmo desempenho e sem danos ao paciente. Estudos mostram existência de lacunas no diagnóstico de infecções fúngicas associado ao diagnóstico da criptococose não abrangente, acesso a testes do diagnóstico rápido (TDR) limitado principalmente em países em via de desenvolvimento que pouco tem sido feito para melhorar o progresso no diagnóstico (7,8).

Em Moçambique as IFI são negligenciadas e os dados são muito escassas. Poucos estudos publicados estimam a incidência anual de meningite criptocócica (MC) em 18.600 casos (70,5 casos por 100.000 pessoas/ano), o país ocupa o terceiro lugar nos países com maior incidência de MC (8,9). O estudo recente publicado usando autópsia diagnóstica completa (ADC) em 284 pacientes falecidos de Moçambique (n=223) e do Brasil (n=61), mostrou que o *Cryptococcus* foi responsável por 16 mortes entre os 163 pacientes HIV positivos (10%; 95 % CI: 6-15%), incluindo quatro mortes maternas (6).

O diagnóstico não invasivo mais acertado no momento da admissão, pode melhorar o desfecho do paciente e contribuir para a redução da mortalidade por criptococose.

## Questões de pesquisa

# Que oportunidades existem para implementar a técnica de diagnóstico *point-of-care* da criptococose no contexto de cuidados e tratamento de criptococose em pacientes HIV?

# Que factores contribuem para o alcance, eficácia, adopção, implementação e manutenção da técnica de diagnóstico *point of care* da criptococose nos hospitais selecionados?

# Revisão bibliográfica

No mundo dados publicados ate 2021 mostraram que a MC associada ao HIV foi responsável por 150.000–200.000 mortes por ano(10). E essas mortes ocorreram principalmente na África Subsaariana, onde a mortalidade foi superior a 70%; 135.900 [95% CI 93.900–163.900] (7,9,10,11). Uma das principais razões para esta alta mortalidade é o atraso no diagnóstico, associado ao acesso limitado à punção lombar e testes de diagnóstico(13). Nesta região, estudos mostram que apesar dos esforços para o tratamento antirretroviral (TARV) há um aumento do peso de infecções invasivas oportunistas incluindo a criptococose (13,14).

A maioria das infecções associadas ao HIV são causadas por *Cryptococcus neoformans*, embora em Botswana, até 30% das infecções sejam por *Cryptococcus gattii*(11).

A MC está entre as doenças mais mal financiadas do mundo. Foi recentemente proposta como uma doença tropical negligenciada (15). Acredita-se que tratamentos prologados com agentes antibacterianos e sem evolução para cura em pacientes HIV com cefaleia e febre, tem provável causa o *Cryptococcus spp* (16,17). A morte por MC é praticamente inevitável na ausência de um tratamento adequado (5).

A prevalência de patógenos fúngicos específicos depende de sua endemicidade. Estudos publicados recentemente mostram que 70% de todos os casos de criptococose em alguns países Africanos parecem apoiar no diagnóstico de HIV e aproximadamente 30% depois do início da TARV (18,19).

A identificação precoce da infecção criptocócica é extremamente crucial (20). O rastreio, tratamento precoce concebido como intervenção mostram-se viável para reduzir o decurso da doença e mortes por MC em doentes HIV/SIDA (14). Na África Subsahariana disponibilizar cuidados de saúde aceitável tem sido um grande desafio, devido a escassez de recursos e testes de diagnóstico são extremamente limitado (7).

O diagnóstico de criptococose é realizado desde o passado a partir da amostra de líquido cefaloraquidiano (LCR) com tinta de China e isolamento da levedura no meio de cultura Sabouraud dextrose. Porém, actualmente a urina é uma amostra altamente desejada para o diagnóstico de criptococose em locais com recursos limitados, poucos médicos e outros especialistas de saúde principalmente nos hospitais de nível primário (18,21), particularmente quando há dificuldades para obtenção de amostras invasivas como sangue ou LCR. *Cryptococcus* spp na urina é indicador de uma criptococose disseminada(22). Em maioria dos casos no País o LCR é colhido por clínicos especialistas.

A amostra de urina tem o potencial de aumentar a oportunidade de diagnóstico precoce de criptococose em muitos ambientes, ela é de fácil obtenção (18,23).

Estudos revelam concordância dos resultados de (sensibilidade e especificidade) usando amostras invasivas (sangue e LCR) quando comparado com amostra não invasiva (urina) no diagnóstico da criptococose (18,24). Saha e colaboradores (24) nos seus achados encontraram um valor preditivo positivo que varia de (82.9, 100%) para tinta-da-China usando amostra de urina o que sugere um bom teste para triagem. Este método de diagnóstico apresenta uma sensibilidade de (85-86,1%) e especificidade 100%, o resultado é obtido no mesmo dia de colheita de amostra.

A OMS (25) e outros estudos publicados por Greene *et al*., 2020 e Stott *et al*., 2021 recomendam o uso da teste de antígeno criptocócico (CrAg) para o rastreio da criptococose em pacientes vivendo com HIV. Porém, há estudos que mostram insustentabilidade do CrAg relacionado a custos em países de baixa renda quando comparado com a tinta-da-China e também os hospitais públicos mostram mais confiança nos meios de diagnóstico mais comumente usado para criptococose, a tinta de China e a cultura (26,27).

A criptococose é uma micose oportunista cuja transmissão se dá através da inalação de propágulos viáveis de Cryptococcus spp. O fungo é encontrado no meio ambiente, em excretos dos pombos, eucaliptos e matéria orgânica em decomposição (13,28). A levedura possui duas variedades reconhecidas *Cryptococcus neoformans* var. *neoformans* (sorotipos A, D e AD), causa acima de 90% das infecções e *Cryptococcus neoformans* var. *gattii* (sorotipos B e C). A variante *grubii* foi recentemente descrita e esta relacionada ao sorotipo A. O fungo tem fase sexual ou teleomórfica que consiste na formação de basidiosporos (29). Na fase parasitária, isto é, nos tecidos o microrganismo aparece como levedura capsulada e algumas vezes com brotamento. O fungo quando inalado atinge primeiro os pulmões onde pode ocorrer latência nos macrófagos alveolares ou proliferação com tropismo para o sistema nervoso central (SNC) ocasionando MC (28,29).

Actualmente a criptococose tem ganhado maior destaque e a sua incidência tem aumentado com o surgimento do HIV. O aumento da incidência de IFIs esta associado ao crescente número de pacientes com factores de risco para estas infecções. As outras condições e factores de risco associados são: a neutropénia, diabetes mellitus e infecção renal, internamento nos cuidados intensivos, uso de imunomoduladores, cirurgia extensa ou queimaduras e intervenções invasivas (12).

Pacientes com criptococose apresentam cefaleia e febre com uma duração média de duas semanas entre o início dos sintomas e os primeiros sinais. Muitos pacientes desenvolvem náuseas, vômitos, diplopia devido a paralisia dos nervos cranianos (NC VI) e redução da acuidade visual relacionada à pressão elevada do LCR. Se não tratados, os sintomas progridem para um estado mental anormal, redução do nível de consciência, convulsões e, finalmente, coma (11).

A doença invasiva clinicamente significativa esta associada a reativação da infecção latente entre indivíduos imunocomprometidos, como pessoas que vivem com HIV, meses a anos após a exposição inicial (13).

Em relação ao tratamento, segundo a Organização Mundial da Saúde (OMS) e o Centro de Controlo e Prevenção de Doenças (CDC) o antifúngico anfotericina B de administração intravenosa e fluconazol oral tem se mostrado eficaz para o tratamento da criptococose (25,30). A OMS recomenda o tratamento da infecção criptocócica assintomática com fluconazol oral e monitoramento rigoroso com vista a reduzir os possíveis efeitos colaterais (25).

# Enquadramento conceptual

O presente estudo será guiado pelo enquadramento conceptual denominado Processo de Adaptação Dinâmica (PAD) que vai apoiar a implementação do método do diagnóstico não invasivo para criptococose nos hospitais selecionados e outro denominado RE-AIM com cinco domínios (Alcance, Eficácia, Adoção, Implementação e Manutenção) para avaliar a implementação **(Figura 2)**. O PAD envolve a identificação de elementos essenciais e características adaptáveis (barreiras e facilitadores) baseadas em evidência, apoiando a implementação com treinamento específico sobre adaptações permitidas ao modelo, monitoramento de fidelidade, suporte e identificação da necessidade e soluções para adaptação do fluxo de trabalho no âmbito de cuidados e tratamento da criptococose (31,32). A fidelidade refere-se a uma avaliação da adesão e competência dos profissionais de saúde. Aderência ao diagnóstico da criptococose com amostra não invasiva que será implementada no presente estudo correspondem as expectativas dos criadores do modelo e será medida como um dos indicadores da intervenção. A habilidade dos profissionais de saúde em usar o modelo é denominado competência. O modelo PAD apresenta quatro fases de implementação (pré-implementação, adaptação, implementação e avaliação), leva em consideração o contexto multinível da entrega de serviços, envolve várias partes interessadas e fornece conhecimento e a retro-informação continua. Durante a intervenção, orienta e fornece “passos” para monitorar a implementação do diagnóstico.


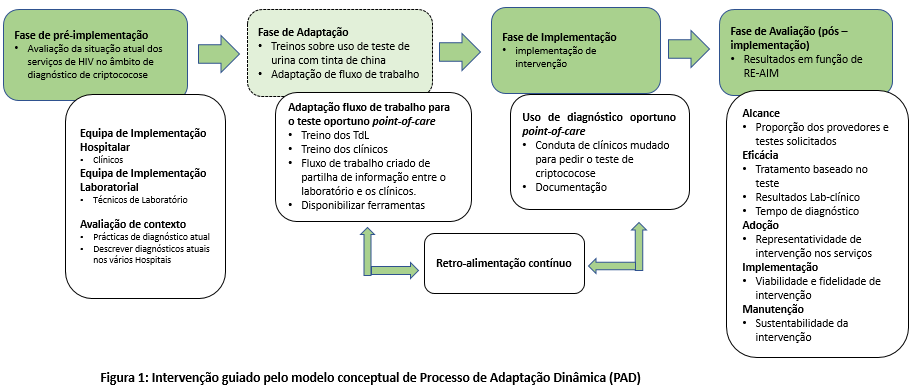


# Metodologia

## Desenho e tipo de estudo

O desenho de estudo é *stepped wedge trial*, com abordagem mista. Estudos *stepped wedge* são prospectivos e de intervenção, a característica principal é o facto de o pesquisador estabelecer a implementação em fases, monitorar as condições da intervenção e permitir avaliar a eficácia do instrumento de intervenção ou da técnica implementada. Este tipo de estudo garante que todos os locais do estudo selecionados eventualmente recebam a intervenção e geralmente traz evidências mais confiáveis. (Veja tabela 1).

**Tabela 1. Desenho de estudo *Stepped-wedge***

| Fases | Meses/2024-2025 | | | | | | | | | | | | | | |
| --- | --- | --- | --- | --- | --- | --- | --- | --- | --- | --- | --- | --- | --- | --- | --- |
|  | 1 | 2 | 3 | 4 | 5 | 6 | 7 | 8 | 9 | 10 | 11 | 12 | 1 | 2 | 3 |
| HCM | C | C | C | C | PI | AD | I | S | S | S | S | S | S | S | S |
| HGM | C | C | C | C | PI | AD |  | I | S | S | S | S | S | S | S |
| HGJM | C | C | C | C | C | PI | AD |  | I | S | S | S | S | S | S |
| HPM | C | C | C | C | C | PI | AD |  |  | I | S | S | S | S | S |
| HPX | C | C | C | C | C | C | PI | AD |  |  | I | S | S | S | S |
| HCC | C | C | C | C | C | C | PI | AD |  |  |  | I | S | S | S |

**C-** Controlo**; PI**-Pré-implementação; **AD**- Adaptação; **I**-Implementação; **S**- Seguimento

A abordagem mista permitirá a triangulação e complementaridade dos achados dando a possibilidade de fortalecer as conclusões de pesquisa (33). A parte quantitativa será do tipo transversal e a qualitativa será genérico. Os dados quantitativos como (número de testes realizados com tinta da China, resultados positivos, resultados negativos, tempo de resposta, nível de instrução académica dos profissionais de saúde, número de profissionais de saúde alocado nos serviços de HIV, número de profissionais treinados, percentagem dos serviços que aderiram a intervenção, número de microscópios existentes) serão obtidos através de questionário administrado aos profissionais de saúde e por observação baseado no guião previamente elaborado. Na parte qualitativa, através de entrevistas semi-estruturadas serão explorados tópicos que abordam (desafios para: o diagnóstico, tratamento da criptococose, e será igualmente avaliado a satisfação dos profissionais de saúde em relação a intervenção).

## Local do estudo

O estudo será realizado em três províncias do sul de Moçambique (Cidade de Maputo, Província de Maputo e Província de Gaza), onde serão selecionados por conveniência os seguintes Hospitais: Hospital Central de Maputo (HCM), Hospital Geral de Mavalane (HGM), Hospital Geral José Macamo (HGJM), Hospital Provincial da Matola (HPM), Hospital Provincial de Xai-Xai (HPX) e Hospital Carmelo de Chokwé (HCC). São hospitais mais diferenciados da zona sul do país.

**Descrição de cada local de estudo:**

O **HCM** é um hospital de referência do país, está localizado na cidade de Maputo, Bairro Central. Está situado entre as Avenidas Eduardo Mondlane, Salvador Allende, Tomás Nduda e Agostinho Neto. É um hospital de nível quaternário e o mais diferenciado do país, com estatuto de Hospital Universitário; O **HGM** é um hospital de nível terciário e está localizado na cidade de Maputo, Distrito Urbano de KaMavota, bairro de Mavalane, na Avenida das Forças Populares de Libertação de Moçambique (FPLM) número798; **O HGJM** é um hospital de nível terciário e está localizado na Avenida da Organização da Unidade Africana (OUA), Distrito urbano [de Nlhamankulu](https://www.africalocal.info/MZ/Maputo/Cidade-De-Maputo/Distrito-Urbano-De-Nlhamankulu/) em Maputo; O **HPM** é um hospital de nível terciário e está localizado na Cidade da Matola, Província de Maputo; **HPX** é um hospital de nível terciário e está localizado na Cidade de Xai-Xai Província de Gaza; **O HCC** é um hospital terciário localizado no distrito de Chokwé, é de referência para doentes de Tuberculose e SIDA na Província de Gaza, é gerido e administrado pelas irmãs da caridade de São Vicente de Paulo, por acordo com o MISAU.

Em termos de capacidade, os hospitais selecionados para o estudo funcionam com capacidade de aproximadamente (239-1500) camas oficiais e possuem os seguintes serviços clínicos: medicina, ortopedia, pediatria, cirurgia, ginecologia-obstetrícia, oncologia, serviço de urgências e reanimação. São hospitais do SNS e são geridos pelo MISAU, importa realçar que Hospital Carmelo, é um hospital de referência para doentes de Tuberculose e SIDA é gerido e administrado pelas irmãs da caridade de São Vicente de Paulo, por acordo com o MISAU. Os hospitais foram selecionados por conveniência (existência de acordo de parceria em pesquisa a muitos anos, possuem serviços de HIV e internamento).

## Período do estudo

- O estudo terá duração de aproximadamente 1 ano e 3 meses com diferentes fases:
- Primeira fase: Avaliação da situação actual: (duração 3 meses):
- Segunda fase: Adaptação (duração 3 meses);
- Terceira fase: Implementação da intervenção em fases guiado pelo PAD (duração 6 meses);
- Quarta fase: Avaliação, sustentabilidade e resultados em função do RE-AIM (duração 3 meses).

## População do estudo

- Médicos, enfermeiros e técnicos de Laboratório dos seguintes hospitais: HCM, HGM, HGJM, HPM, HPX e HCC.

## Amostra e amostragem

**Amostra para a componente qualitativa do estudo**

O tamanho de amostra será determinado com base em dois princípios: estudos precedentes de implementação de técnicas nos hospitais e saturação teórica. A amostra em estudos precedentes a este varia de “24 -30 profissionais de saúde”. Uma vez que Moçambique tem escassez de profissionais de saúde determinou-se que serão envolvidos no estudo todos profissionais disponíveis (médicos, enfermeiros e técnicos de laboratório) em serviço na medicina e laboratório de microbiologia dos hospitais selecionados.

**Profissionais por serem envolvidos**

HCM: 8 clínicos nas medicinas e 2 técnicos de laboratório;

HGM: 4 clínicos nas medicinas e 2 técnicos de laboratório;

HGJM: 4 clínicos nas medicinas e 2 técnicos de laboratório;

HPM: 3 clínicos nas medicinas e 2 técnicos de laboratório;

HPX: 3 clínicos nas medicinas e 2 técnicos de laboratório;

HCC: 2 clínicos nas medicinas e 2 técnicos de laboratório.

**Amostragem**

A amostragem será não probabilística onde os participantes serão selecionados de forma intencional e os locais de estudo (hospitais) foram selecionados por conveniência. O envolvimento dos hospitais selecionados deve-se a existência de acordo de parceria em pesquisa a muitos anos com os mesmos, sendo que possuem igualmente serviços de medicina associado a TARV e internamento que é uma grande vantagem para implementação do presente estudo.

O investigador não terá contacto com os pacientes, não vai recrutar os pacientes. O clínico é que decide sobre a que pacientes pedir a amostra da urina para diagnóstico de criptococose. O estudo vai fornecer ao sistema nacional de saúde um recurso para o diagnóstico da criptococose.

**Descrição da técnica a ser implementada**

A técnica da tinta da China em urina é uma técnica quantitativa, financeiramente de baixo custo, de fácil execução e conservação. Realizada a partir da amostra não invasiva (urina) de fácil obtenção. Uma gota de urina de aproximadamente 2ml é depositada na lâmina microscópica, de seguida adiciona-se uma gota da tinta da China e cobre se com a lamela. Resultado é obtido em aproximadamente 15 minutos, permite observar leveduras capsuladas sugestivas de *Cryptococcus* spp.

## 7.6. Critério de inclusão e exclusão

**7.6.1. Critério de inclusão**

- Serão incluídos técnicos de laboratório afectos ao sector de Microbiologia, médicos e enfermeiros afectos aos serviços de medicina de cada hospital selecionada.

**7.6.2. Critério de exclusão**

- Serão excluídos todos profissionais de saúde que não estão diretamente ligados ao diagnóstico microbiológico e ao cuidado de pacientes adultos com HIV.

**7.8. Procedimentos, instrumentos de recolha e análise de dados**

Os dados quantitativos serão colhidos pelo investigador com base no guião de observação e questionário acoplado a plataforma REDCap carregada no *tablet*. Serão colhidas informações demográficas dos profissionais, serão listados e descritos todos equipamentos e materiais actualmente em uso para cuidados e tratamento da criptococose, incluído a recolha de informação relacionada a infraestrutura de cada unidade sanitária

Será igualmente feita a descrição dos diagnósticos de criptococose actualmente realizadas em cada hospital envolvido no estudo e identificar barreiras e facilitadores para implementação da técnica de diagnóstico da criptococose com amostra da urina.

Com base no quadro conceptual PAD será feita a implementação do diagnóstico laboratorial da criptococose nos seis hospitais selecionados, será realizado treino, criação de fluxo de partilha de informação entre técnicos de laboratório e clínicos, será padronizado a definição de caso de criptococose e facultadas as ferramentas guias de diagnóstico e tratamento.

Durante a implementação os clinicos irão solicitar amostra de urina a pacientes elegíveis e execução pelos técnicos de laboratorio treinados, será feira a monitoria, documentação da intervenção e garantida a retro - informação continua entre os clinicos e técnicos de laboratório.

A implementação será avaliada e os resultados com base no quadro conceptual RE-AIM de cinco domínios alcance (abrangência), eficácia, adoção (aceitabilidade e representatividade), implementação e manutenção (sustentabilidade) da intervenção nos hospitais.

**Análise de dados qualitativos**

O investigador irá colher dados qualitativos através de entrevistas semi-estruturadas administradas aos profissionais de saude, com duração de 25 minutos cada e gravadas no gravador digital.

A análise de dados qualitativos será baseada no *Software Nvivo* versão 12. Será realizada uma análise temática, com espaço para o surgimento de hipóteses e novas teorias em relação ao tema.

As entrevistas gravadas serão transcritas em *Word* sem alterar a sua originalidade e classificadas em categorias previamente criadas. Os participantes serão anonimizados usando números e iniciais dos seus nomes. O controlo de qualidade das transcrições será realizado confrontando se o que foi escrito com o áudio da entrevista para garantir que não houve omissão ou acréscimo de informação, e garantido assim a originalidade da informação.

O *Software NVivo* será usado para auxiliar na organização e controlo dos dados e o estudo terá igualmente suporte dos quadros conceptual PAD e RE-AIM. As passagens de texto serão agrupadas em temas de acordo com os quadros conceptual.

Nesta componente qualitativa serão gerados os seguintes construtos:

1. Conhecimento dos profissionais de saúde sobre o peso da criptococose no seu hospital e no Pais;
2. Conhecimento dos profissionais de saúde sobre métodos do diagnóstico da criptococose no seu hospital;
3. Perceção de barreiras no âmbito da triagem, diagnóstico e tratamento da criptococose no seu hospital;
4. Sondagem: Oportunidades para o novo diagnóstico da criptococose no seu hospital.

**Análise de dados quantitativos**

Os dados quantitativos serão analisados no pacote estatístico *R studio*. Serão feita análises descritivas uni-variadas (será calculada a média ou mediana de idade, desvio padrão e a variância), frequências percentuais das seguintes variáveis: testes solicitados, médicos/técnicos de laboratório que aderiram a intervenção, qui-quadrado para verificar associações entre a intervenção/teste e as variáveis (nível de instrução do profissional, disponibilidade do teste, gravidade da doença e sexo) e regressão logística para estimar a relação entre aderência ao teste e factores gravidade da doença, sobrecarga do trabalho na US/laboratório, nível de instrução do profissional, turno, sexo, disponibilidade de serviços de laboratório.

Será usada a escala de Likert para avaliar o nível de satisfação de todos abrangidos pela intervenção (médicos e técnicos de laboratório) durante o período do estudo. E serão calculadas as frequências das informações obtidas.

Os resultados serão descritos, apresentados igualmente em tabelas e gráficos, usando o programa Microsoft Excel. O valor do p<0.05 será considerado significativo.

## 7.9. Estudos

O estudo prevê três sub-estudos:

# Práticas e desafios existentes no diagnóstico laboratorial da Criptococose em seis hospitais das três Províncias de Sul de Moçambique.

Trata-se de um estudo do tipo transversal com abordagem mista. A abordagem qualitativa será genérica onde será feita a avaliação da situação actual antes da implementação da intervenção (Figura 2: *Fase de Pré-implementação*) de modo a identificar elementos do contexto social, sanitário, administrativo e estrutural que podem favorecer ou não a intervenção no contexto local.

A avaliação da situação actual será realizado em seis hospitais (HCM, HGM, HGJM, HPM, HPX e HCC) durante três meses. Amostragem será não probabilística intencional em relação aos participantes e por conveniência em relação aos locais de estudo.

Os dados para este estudo serão obtidos por questionários semi-estruturados (Anexo I, II e IV) e entrevistas (anexo III) a profissionais de saúde de cada hospital (Clínicos do serviço de medicina e Técnicos de Laboratório de microbiologia); e será feita a listagem de equipamento, material existente e a descrição dos diagnósticos de criptococose actualmente realizadas em cada hospital envolvida no estudo e identificar barreiras e facilitadores para implementação da técnica de diagnóstico da criptococose com amostra da urina.

A análise de dados será feita com base no pacote estatístico R *studio*, onde serão feitas as seguintes análises: análise descritiva (calcular frequências, média ou mediana, desvio padrão e variância); e qui-quadrado para comparar frequências. Os resultados serão descritos, apresentados igualmente em tabelas e gráficos. O valor do p<0.05 será considerado significativo.

# Implementar a técnica de diagnóstico laboratorial da Criptococose pelo método de tinta de China em urina como *point of care* nos hospitais selecionados de três Províncias de sul de Moçambique.

Trata-se de um estudo *stepped wedge trial*. Amostragem será não probabilística por conveniência em relação aos locais de estudo e intencional em relação aos participantes.

O estudo será realizado em seis hospitais (HCM, HGM, HGJM, HPM, HPX e HCC) durante um período de seis meses. A *Fase de Adaptação* será o início da iteração com o novo teste, os profissionais de saúde (clínicos em serviço de medicina e técnicos de laboratório afectos ao sector de microbiologia) adaptam-se ao fluxo de trabalho associado á intervenção (Figura 2). Durante os três meses de adaptação, o investigador irá realizar um treino sobre cuidados, diagnóstico e tratamento da criptococose usando tinta-da-China na urina (5 a 20 ml da amostra) com profissionais de saúde selecionados intencionalmente, serão facultadas as ferramentas guias de diagnóstico e será estabelecido um fluxo de trabalho de partilha de informação entre o laboratório e os clínicos.

O clínico é que decide sobre a que pacientes pedir a amostra da urina para diagnóstico de criptococose e também decidi que informação clínica partilha com o laboratório de acordo com cada situação. Vamos fornecer ao sistema nacional de saúde um recurso para o diagnóstico da criptococose, em vez do tratamento empírico quando o de rotina não esta disponível. Portanto, o clinico esta livre de solicitar teste de rotina ou experimental. O clinico se decidir solicitar a amostra de urina para o diagnostico da criptococose o laboratório irá dar o *feedback* segundo a solicitação do clinico para dar seguimento do paciente.

A *implementação* do diagnóstico da criptococose com tinta-da-China usando amostra não invasiva urina nos locais selecionados, será durante 6 meses. Nesta *fase de implementação* os profissionais de saúde afectos ao serviço de medicina com conhecimento da intervenção (novo teste para criptococose) irão solicitar a sua realização aos colegas do laboratório previamente treinados para a execução. Ao longo desta fase (Figura 2) o monitoramento da conduta dos clínicos em relação ao uso de novo diagnóstico ocorrerá e a documentação de sua implementação será capturada (Anexo V).

A análise de dados será feita com base no pacote estatístico R *studio.* Serão calculadas as f**requência**s das variáveis (número de testes solicitados com e sem feedback, número de profissionais treinados) e será feita a comparação das frequências usado o **Qui-quadrado**.

Será determinada **a correlação** entre (Número de profissionais treinados e número de testes solicitados); Serão estimados os **“Odds Ratio” (OR)** usado **a regressão**: serviços abrangidos/melhorados (variável dependente) e variáveis independentes: profissional treinado, anos de prática, nível de instrução do profissional; idade, sexo.

**3. Avaliação da implementação com base no RE-AIM da técnica do diagnóstico laboratorial da criptococose pelo método de *tinta de China* em urina como *point of care* no sul de Moçambique**

Trata-se de um estudo quantitativo transversal onde pretende-se a aferir a contribuição do diagnóstico introduzido (efeito da intervenção) nos seis hospitais (HCM, HGM, HGJM, HPM, HPX e HCC) durante três meses. Amostragem será não probabilística intencional em relação aos participantes (profissionais de saúde) e por conveniência em relação aos locais de estudo.

Na *Fase de Avaliação* da implementação (Pós-implementação), durante três meses a implementação será avaliada usando o quadro conceptual **RE-AIM** com cinco domínios **alcance** (abrangência), **eficácia, adoção** (aceitabilidade e representatividade), **implementação** e **manutenção** (sustentabilidade) da intervenção nos hospitais. A recolha de dados será baseada em questionários e guiões padronizados previamente elaborados acoplados na plataforma *REDCap* carregada no *tablet.*

- Alcance será calculada através da proporção de indivíduos abrangidos pela intervenção (clínicos e técnicos de laboratório).
- A eficácia será avaliada através de verificação de resultados laboratoriais e clínicos, tempo de diagnóstico e satisfação dos profissionais de saúde.

O estudo contempla uma avaliação no início (*base-line*) pré-implementação e no final pós-implementação onde será verificado nos dois momentos os resultados laboratoriais e clínicos, tempo de diagnóstico e satisfação dos profissionais de saúde. Portanto a eficácia da intervenção será avaliada antes e depois da implementação. Serão consultados as recomendações da OMS, onde recomendam triagem e tratamento para a prevenir complicações por uma infecção cryptococica. O teste a ser implementado vai contribuir para o início do tratamento no mesmo dia da consulta e baseado no diagnóstico etiológico.

- A adoção será explorada através da representatividade dos serviços que adotaram a implementação incluindo o fluxo de amostra, processamento pelo laboratório e feedback/ retro informação, uso de guiões elaborados e disponibilizados durante a intervenção.
- Será igualmente usada a escala de Likert para analisar a satisfação de todos profissionais de saúde abrangidos pela intervenção durante o período de estudo (clínicos e técnicos de laboratório).
- Durante a implementação será avaliada a fidelidade dos profissionais de saúde em relação ao diagnóstico, cuidados e tratamento da criptococose baseada na observação documentada.
- A manutenção (sustentabilidade) será avalida baseda na simplicidade da intervenção (técnica do diagnóstico laboratorial da criptococose usando a urina), do fluxo do trabalho e do custo do teste implementado.

Os dados obtidos neste estudo serão introduzidos no pacote estatístico R *studio,* onde serão realizadas as seguintes análises: Análise descritiva (calcular frequências, média e variância), Qui-quadrado para comparar frequências e regressão multivariada estimar os “Odds Ratio” (OR).

## Variáveis e Plano de análise de dados

| **Objectivo** | **Variáveis** | **Plano de Análise de dados** |
| --- | --- | --- |
| Analisar as práticas e os desafios existentes no diagnóstico laboratorial da criptococose nos hospitais HCM, HGM, HGJM, HPM, HPX e HCC. | Nível do hospital, nível de instrução, nacionalidade, anos de prática, sexo, idade, estado civil, tipos de testes solicitados, disponibilidade dos testes, número de amostras testadas, participação no controlo de qualidade, disponibilidade do microscópio, disponibilidade da centrífuga, disponibilidade do reagente, fluxo de trabalho por turno. | Análise descritiva calcular a **frequência** de (testes solicitados, amostras testadas, tipo de teste, sexo, anos de pratica, idade) e média, desvio padrão, variância; **Qui-quadrado** estabelecer relação entre o nível de instrução, sexo e o tipo do teste solicitado  **Teste t independente**: relação entre o fluxo de trabalho por turno e tipo de teste, número de testes solicitados com e sem *feedback*.  **Dados qualitativos, Nvivo** versão 12: análise de conteúdos |
| Implementar a técnica de diagnóstico laboratorial da Criptococose pelo método de tinta de China em urina como *point of care* nos hospitais selecionados. | Nível do hospital, nível de instrução do Profissional de saúde, profissional treinado, número de testes solicitados, tipo de testes solicitados, número de amostras testadas, tempo de resposta, uso de guiões facultados, completude dos dados preenchidos. | **Frequência**: solicitações de teste, resultados laboratoriais partilhados pelos técnicos, **Qui-quadrado** (relação solicitação do teste e feedback do lab); **Correlação:** (Número de profissionais treinados e número de testes solicitados); R**egressão multivariada** estimar os “Odds Ratio” (OR) da relação entre a solicitação do enxame VD & variáveis independentes: nível de instrução do profissional, anos de prática, nível do hospital, turno, sobrecarga do trabalho na clínica/laboratório, idade do profissional, sexo, uso de guiões facultados. |
| Avaliar a implementação do método de tinta de China como o diagnóstico laboratorial *point of care* nos hospitais selecionados, com base no quadro conceitual de RE-AIM. | Número de serviços e número de provedores que aderiram a implementação, idade, sexo, , número de amostras testadas, disponibilidade do teste, início de tratamento após o teste positivo. | **Frequência** (profissionais que aderiram a intervenção, amostras testadas, serviços que aderiram a intervenção, idade e sexo. **Qui-quadrado** comparar as frequências Ex: (relação entre características dos profissionais e o tipo do teste solicitado);  **Regressão multivariada** estimar os “Odds Ratio” (OR) da relação entre a aderência ao teste (PS) e as variáveis: gravidade da doença, idade do profissional, sexo, US, disponibilidade dos serviços no laboratório e *feedback* |

# Limitações do estudo

Foram identificadas algumas limitações, nomeadamente:

- A natureza do estudo *stepped wedge* que é longo e de seguimento dos participantes tem risco de perda dos mesmos introduzindo viés na análise dos desfechos;

Para minimizar este facto serão envolvidos no treino todos os clínicos do serviço de medicina e técnicos de laboratório do sector de microbiologia e serão realizadas as análises transversais dos desfechos.

- A recolha de dados por questionário auto – administrado é provável limitação porque o participante pode responder para agradar a instituição e os gestores da mesma, e o uso de entrevista para a recolha de dados qualitativos há risco de os participantes responder de maneira que acham que o investigador quer ouvir;

Para minimizar esse facto os questionários serão anónimos, o investigador estará presente no acto de administração do questionário e durante as entrevistas para clarificar o entrevistado em relação as questões.

# Considerações éticas

Os dados colhidos para este estudo não terão informações que permitem identificar os participantes envolvidos, serão codificados e anonimizados. A participação será mediante a assinatura do consentimento informado, a ligação entre o código e o nome dos participantes só será conhecida pelos investigadores. A confidencialidade será mantida em todos os momentos da pesquisa, os dados serão usados apenas para este estudo e nunca para outros fins.

O estudo não irá alterar o tipo de amostra de diagnóstico de criptococose em vigor nos hospitais envolvidos. O estudo ira demonstrar aos clínicos e técnicos de laboratórios uma alternativa de diagnóstico de criptococose com amostra não invasiva e fácil de obter. Os pacientes com diagnóstico positivo para criptococose serão tratados de acordo com as normas do Ministério da Saúde (MISAU) em vigor.

Os investigadores declaram que vão cumprir com as boas práticas de ética na pesquisa. O estudo será submetido as Direcções dos Hospitais selecionados e ao Comité Institucional de Bioética em Saúde da Faculdade de Medicina/Hospital Central de Maputo (CIBS FM & HCM). E será igualmente revisto e aprovado pelo Comite Nacional de Bioética para a Saúde (CNBS).

## Potenciais riscos e como estes serão minimizados

O estudo prevê riscos mínimo para os participantes envolvidos no estudo, como desconforto, cansaço e ansiedade. Para mitigar o risco durante o treino será enfatizada a importância de garantir e fortalecer a partilha de informação entre os clínicos e técnicos de laboratório e será igualmente fornecido uma linha de comunicação de acesso aos investigadores do estudo para esclarecimento das suas dúvidas, apoio e resolução das suas preocupações.

## Consentimento informado

O processo do consentimento informado começa com inclusão no pré-estudo, quando a equipe do estudo explica toda a informação pertinente relacionada com o estudo. O pessoal do estudo irá explicar para cada um dos potenciais participantes, a natureza do estudo, a sua finalidade, os procedimentos envolvidos, a duração prevista, os potenciais riscos e benefícios envolvidos, e qualquer desconforto que possa estar associado a participação no estudo. Veja o consentimento informado no apêndice da página 38.

## Confidencialidade

A confidencialidade das informações recolhidas é extremamente fundamental. A equipa de pesquisa será treinada para aderir, cumprir, seguir as normas rigorosamente e proteger a confidencialidade dos participantes.

Serão usadas as seguintes medidas para proteger a confidencialidade das informações prestadas:

- Durante a capacitação, será enfatizada á equipa de pesquisa, a importância de manter o sigilo;
- As entrevistas serão realizadas em ambientes privados;
- Os termos do consentimento informado assinados serão arquivados num cacifo de arquivos trancado separadamente dos outros dados do estudo;
- Os detalhes de contactos dos participantes serão gravados de forma segura em computadores protegidos por uma senha separadamente dos outros dados do estudo;
- Não haverá registo de nomes em nenhum termo de recolha de dados, para o efeito, usaremos números de identificação únicos (ID`s) que serão atribuídos a cada participante;
- Informação colhida de forma não intencional que for identificada nas gravações será editada;
- Os dados serão inseridos directamente a um dispositivo protegido por senha.
- Os dados serão regularmente inseridos a um servidor seguro.
- Apenas um número restrito do pessoal do estudo, cujas funções requerem acesso à informação com identificação (ou seja, o termo de consentimento informado assinado, informações de contacto, lista principal que relaciona os nomes dos participantes com o número do estudo ID) é que terá acesso a este tipo de dados;
- Os participantes não serão identificados por seus próprios nomes em qualquer relatório ou publicação resultante dos dados da pesquisa.

As informações serão recolhidas através da plataforma *REDCap* (*Research Electronic Data Capture***)** carregada no *tablet*. Todas as cópias de dados, arquivos electrónicos incluindo as transcrições no *Microsoft Word* e bancos de dados serão arquivados de forma segura na Faculdade de Medicina por um período de cinco anos após a conclusão da pesquisa.

## Potenciais benefícios

Os profissionais de saúde terão uma oportunidade de serem treinados para o diagnóstico da criptococose com uma técnica financeiramente de baixo custo, que usa uma amostra não invasiva (urina) de fácil obtenção. Uma vez capacitados os profissionais de saúde terão possibilidade de recorrer a este teste para o rastreio laboratorial dos casos suspeitos de criptococose e tratem baseado no diagnóstico etiológico não apenas clinico (empírico).

- 1. Declaração de conflitos de interesse

Os investigadores declaram não haver conflito de interesse no presente estudo, nem benefícios monetários resultantes da condução do estudo.

- 1. Resultados esperados

Espera-se que:

- O estudo traga evidências das práticas e os desafios existentes no diagnóstico laboratorial da Criptococose;
- Os profissionais de saúde estejam capacitados, oportunidade de diagnóstico aumentado e início do tratamento mais próximo do momento da admissão com vista a melhorar o desfecho dos pacientes;
- A técnica de tinta de China para triagem laboratorial *point-of-care* esteja implementado e funcional;
- Os dados possam contribuir em informação ao MISAU e que haja oportunidade para fazer advocacia de modo que seja priorizado estratégias de diagnóstico de criptococose menos dispendioso, maximizar o controlo e prevenção desta doença negligenciada que juntamente com o HIV, estão devastando a saúde e os recursos socioeconômicos no Pais.
  1. Disseminação de resultados

Os resultados serão apresentados primeiro nos locais de recolha de dados (hospitais), a seguir no programa de Doutoramento, nas conferências científicas e finalmente publicados em revistas científicas.

# Cronograma do estudo

| **ACTIVIDADES** | **MESES/2022 – 2026** | | | | | | | | | | | |
| --- | --- | --- | --- | --- | --- | --- | --- | --- | --- | --- | --- | --- |
|  | **02/2022** | **03-07/2022** | **08-12/2022** | **01-03/2023** | **04-12/2023** | **01/2024-03/2024** | **04/2024-12/2024** | **01-06/2025** | **07-09/2025** | **10/2025-11/2025** | **12/2025-03/2026** | **04/2026** |
| Revisão bibliográfica |  |  |  |  |  |  |  |  |  |  |  |  |
| Preparação do protocolo |  |  |  |  |  |  |  |  |  |  |  |  |
| Submissão do Projecto ao Programa de Doutoramento |  |  |  |  |  |  |  |  |  |  |  |  |
| Defesa do projecto de pesquisa |  |  |  |  |  |  |  |  |  |  |  |  |
| Submissão do Projecto ao Comité Institucional de Bioética em Saúde da FM & HCM e CNBS |  |  |  |  |  |  |  |  |  |  |  |  |
| Avaliação da situação actual (Fase de pré-implementação) |  |  |  |  |  |  |  |  |  |  |  |  |
| Fase de adaptação e implementação da intervenção |  |  |  |  |  |  |  |  |  |  |  |  |
| Submissão do manuscrito I |  |  |  |  |  |  |  |  |  |  |  |  |
| Inglês e Ética |  |  |  |  |  |  |  |  |  |  |  |  |
| Fase de avaliação da implementação e Submissão do manuscrito II |  |  |  |  |  |  |  |  |  |  |  |  |
| Submissão do manuscrito III |  |  |  |  |  |  |  |  |  |  |  |  |
| Especialidade e Elaboração da proposta de financiamento |  |  |  |  |  |  |  |  |  |  |  |  |
| Sabática de escrever |  |  |  |  |  |  |  |  |  |  |  |  |
| Defesa da tese |  |  |  |  |  |  |  |  |  |  |  |  |

# Equipa do estudo

José Langa, Residente do programa de Doutoramento em Biociências e Saúde Pública na Faculdade de Medicina, UEM, Investigador principal;

Professor Mohsin Sidat, Faculdade de Medicina, UEM, Supervisor;

Professor Troy D. Moon, Tulane University School of Public Health and Tropical Medicine-USA, Supervisor;

Professor Jahit Sacarlal, Faculdade de Medicina, UEM_Supervisor.

Ponto focal (Clínico e técnico de laboratório) – HCM;

Ponto focal (Clínico e técnico de laboratório) – HGM;

Ponto focal (Clínico e técnico de laboratório) – HGJM;

Ponto focal (Clinico e técnico de laboratório) – HPM;

Ponto focal (Clinico e técnico de laboratório) – HPX;

Ponto focal (Clinico e técnico de laboratório) – HCC.

- 1. Papel de cada integrante da equipe

1. Investigador principal: Facilitar o treino, recolha de dados, análise e redação do relatório;
2. Pontos focais clínicos de cada US: Gestão de dados na US e partilha com o investigador principal;
3. Técnicos de laboratório: processamento de amostras
4. Supervisores/Mentores: Orientar o investigador na condução da pesquisa, redação do relatório e identificar oportunidades para divulgação dos resultados da pesquisa.

# Orçamento

| **Iten** | **Quantidade** | **Custo unitário (Mt)** | **Total (Mt)** |
| --- | --- | --- | --- |
| **Material de escritório** | | | |
| Lápis | 40 | 20,00 | 800,00 |
| Borracha | 40 | 20,00 | 800,00 |
| Afiador | 40 | 20,00 | 800,00 |
| Esferográfica | 40 | 25,00 | 1.000,00 |
| Resma | 25 | 400,00 | 10.000,00 |
| Impressão |  |  | 30.000,00 |
| Fotocópias |  |  | 22.000,00 |
| Gravador | 1 |  |  |
| **Subtotal 1** | | | **65.400,00** |
|  | | | |
| **Material do Laboratório** | | | |
| Tinta da China (30ml) | 6kits | 400,00 | 1.600,00 |
| Lâminas microscopicas | 12cx | 975,00 | 11.700,00 |
| Lamelas | 12cx | 1410,00 | 16.920,00 |
| Luvas tamanho médio sem pó |  |  | 20.550,00 |
| **Subtotal 2** | | | **50.770,00** |
|  | | | |
| **Pessoal de apoio** | | | |
| Profissionais de saúde | 6 | 20.000,00 | 120.000,00 |
| **Subtotal 3** | | | **120.000,00** |
|  | | | |
| **Viagens e comunição** | | | |
| Viagens comunicação | | | 80.000,00 |
| **Subtotal 4** | | | **80.000,00** |
|  | | | |
| **Total** | | | **316.170,00** |
| **Imprevisto 10%** | | | **31.617.00** |
| **Total com 10% de imprevistos** | | | **347.787.00** |

# Referências Bibliográficas

1. INSIDA. Inquerito nacional sobre o impacto do HIV e SIDA em Mocambique. 2021.

2. World Health Organization (WHO). The Health of the people: The African Regional health report. 2006.

3. UNICEF. Monitoring and statistics. In 2005.

4. World Health Organization (WHO). The world health report 2004—changing history. Geneva. 2004.

5. French N, Gray K, Watera C, Nakiyingi J, Lugada E, Moore M, et al. Cryptococcal infection in a cohort of HIV-1-infected Ugandan adults: AIDS. 2002 May;16(7):1031–8.

6. Hurtado JC, Castillo P, Fernandes F, Navarro M, Lovane L, Casas I, et al. Mortality due to Cryptococcus neoformans and Cryptococcus gattii in low-income settings: an autopsy study. Sci Rep. 2019 Dec;9(1):7493.

7. Petti CA, Polage CR, Quinn TC, Ronald AR, Sande MA. Laboratory Medicine in Africa: A Barrier to Effective Health Care. Clinical Infectious Diseases. 2006 Feb 1;42(3):377–82.

8. Rajasingham R, Smith RM, Park BJ, Jarvis JN, Govender NP, Chiller TM, et al. Global burden of disease of HIV-associated cryptococcal meningitis: an updated analysis. The Lancet Infectious Diseases. 2017 Aug;17(8):873–81.

9. Sacarlal J, Denning D. Estimated Burden of Serious Fungal Infections in Mozambique. JoF. 2018 Jun 23;4(3):75.

10. Stott KE, Loyse A, Jarvis JN, Alufandika M, Harrison TS, Mwandumba HC, et al. Cryptococcal meningoencephalitis: time for action. The Lancet Infectious Diseases. 2021 Sep;21(9):e259–71.

11. Limper AH, Adenis A, Le T, Harrison TS. Fungal infections in HIV/AIDS. The Lancet Infectious Diseases. 2017 Nov;17(11):e334–43.

12. Jarvis JN, Bicanic T, Loyse A, Namarika D, Jackson A, Nussbaum JC, et al. Determinants of Mortality in a Combined Cohort of 501 Patients With HIV-Associated Cryptococcal Meningitis: Implications for Improving Outcomes. Clinical Infectious Diseases. 2014 Mar 1;58(5):736–45.

13. WHO. Guidelines for the diagnosis, prevention and management of cryptococcal disease in HIV-Infected adults, adolestents and childrens. 2018.

14. Greene G, Lawrence DS, Jordan A, Chiller T, Jarvis JN. Cryptococcal meningitis: a review of cryptococcal antigen screening programs in Africa. Expert Review of Anti-infective Therapy. 2021 Feb 1;19(2):233–44.

15. Molloy SF, Chiller T, Greene GS, Burry J, Govender NP, Kanyama C, et al. Cryptococcal meningitis: A neglected NTD? Zunt JR, editor. PLoS Negl Trop Dis. 2017 Jun 29;11(6):e0005575.

16. Ordi J, Castillo P, Garcia-Basteiro AL, Moraleda C, Fernandes F, Quintó L, et al. Clinico-pathological discrepancies in the diagnosis of causes of death in adults in Mozambique: A retrospective observational study. Moreira J, editor. PLoS ONE. 2019 Sep 6;14(9):e0220657.

17. Bongomin F, Gago S, Oladele R, Denning D. Global and Multi-National Prevalence of Fungal Diseases—Estimate Precision. JoF. 2017 Oct 18;3(4):57.

18. Jarvis JN, Percival A, Bauman S, Pelfrey J, Meintjes G, Williams GN, et al. Evaluation of a Novel Point-of-Care Cryptococcal Antigen Test on Serum, Plasma, and Urine From Patients With HIV-Associated Cryptococcal Meningitis. Clinical Infectious Diseases. 2011 Nov 15;53(10):1019–23.

19. Jarvis JN, Meintjes G, Harrison TS. Outcomes of cryptococcal meningitis in antiretroviral naive and experienced patients in South Africa. J Infect. 2010;496–8.

20. Chastain DB, Henao-Martínez AF, Franco-Paredes C. Opportunistic Invasive Mycoses in AIDS: Cryptococcosis, Histoplasmosis, Coccidiodomycosis, and Talaromycosis. Curr Infect Dis Rep. 2017 Oct;19(10):36.

21. Sara. Invetario Nacional. 2018.

22. Kiertiburanakul S, Sungkanuparph S, Buabut B, Pracharktam R. Cryptococcuria as a Manifestation of Disseminated Cryptococcosis and Isolated Urinary Tract Infection.

23. Pinto Junior VL, Galhardo MCG, Lazéra M, Wanke B, Reis RS, Perez M. Criptococose associada à Aids: a importância do cultivo da urina no seu diagnóstico. Rev Soc Bras Med Trop. 2006 Apr;39(2):230–2.

24. Saha DC, Xess I, Biswas A, Bhowmik DM, Padma MV. Detection of Cryptococcus by conventional, serological and molecular methods. Journal of Medical Microbiology. 2009 Aug 1;58(8):1098–105.

25. World Health Organization. Rapid advice Diagnosis, Prevention anD ManageMent of CryPtoCoCCal Disease in Hiv-infeCteD aDults, aDolesCents anD CHilDren. 2011.

26. Deiss R, Loreti CV, Gutierrez AG, Filipe E, Tatia M, Issufo S, et al. High burden of cryptococcal antigenemia and meningitis among patients presenting at an emergency department in Maputo, Mozambique. Kufa T, editor. PLoS ONE. 2021 Apr 26;16(4):e0250195.

27. Rajasingham R, Wake RM, Beyene T, Katende A, Letang E, Boulware DR. Cryptococcal Meningitis Diagnostics and Screening in the Era of Point-of-Care Laboratory Testing. Kraft CS, editor. J Clin Microbiol [Internet]. 2019 Jan [cited 2021 Aug 6];57(1). Available from: https://journals.asm.org/doi/10.1128/JCM.01238-18

28. Murray PR Ken and Pfaller, Michael. Microbiologia Medica. 6th ed. 2010.

29. Jawetz, Melnick, & Adelberg’s. Medical Microbiology. 26th Edition. 2013.

30. Centers for Disease Control and Prevention (CDC). Cryptococcal Screening Program Training Manual for Healthcare Providers. 2012.

31. Aarons GA, Green AE, Palinkas LA, Self-Brown S, Whitaker DJ, Lutzker JR, et al. Dynamic adaptation process to implement an evidence-based child maltreatment intervention. Implementation Sci. 2012 Dec;7(1):32.

32. Glasgow RE, Vogt TM, Boles SM. Evaluating the public health impact of health promotion interventions: the RE-AIM framework. Am J Public Health. 1999 Sep;89(9):1322–7.

33. Schoonenboom J, Johnson RB. How to Construct a Mixed Methods Research Design. Köln Z Soziol. 2017 Oct;69(S2):107–31.

# APÊNDICES E ANEXOS

**Folha de Informações ao Participante e Termo de Consentimento Informado e esclarecido**

**(PS)**

**PARTE I**

**Título do protocolo**

**Implementação da técnica de tinta da China em urina para triagem laboratorial *point-of-care* da Criptococose em pacientes adultos infectados por HIV no sul de Moçambique**

**Versão do protocolo**: 04, Outubro 2023

**Versão do Consentimento/Folha de Informação ao participante**: 04, Outubro 2023

**Investigador principal**: José Langa^1^;

**Co-Investigadores**: Mohsin Sidat^1^ ;Jahit Sacarlal^1^ e Troy D. Moon^2^

**Filiação dos Investigadores**

1.Faculdade de medicina - Universidade Eduardo Mondlane

2. Tulane University School of Public Health and Tropical Medicine-USA

**Nome do Financiador/ Patrocinador**

*Partnership for Research in Implementaion Science – Mozambique (PRISM)* uma colaboração entre a Universidade Eduardo Mondlane (UEM) e Tulane University School of Public Health and Tropical Medicine-USA com financiamento de Fogarty International Center de National Institutes of Health de Estados Unidos da América (EUA).

NB: Por favor solicite sempre que necessário um membro da equipa do estudo que lhe explique o que não tiver entendido durante a leitura deste formulário de consentimento informado e não assine sem ter respostas a todas as suas perguntas.

**Introdução**:

A criptococose é uma infecção oportunista e uma das principais causas de consulta e internamento de adultos vivendo com HIV na África Subsaariana. O diagnóstico precoce pode evitar complicações por esta infecção.

O diagnóstico clássico de criptococose é realizado através da pesquisa do microorganismo no líquido cefalorraquidiano (LCR) ou sangue. Actualmente as amostras invasivas são substituídas por amostras alternativas não invasivas, fáceis de obter e sem danos ao paciente.

O presente estudo será conduzido nos Hospitais: Central de Maputo, Geral de Mavalane, Geral José Macamo, Provincial da Matola, Provincial de Xai-Xai e Carmelo de Chokwé.

O Boletim de consentimento informado expõem o motivo da pesquisa. Nele está explicado os procedimentos, possíveis riscos e benefícios para si, se concordar em participar no estudo

**Justificação da pesquisa**

A criptococose assume ultimamente papel de destaque entre as doenças que afectam a saúde e o bem-estar da população em todo mundo. A criptococose é uma doença oportunista negligenciada associada ao aumento de número de consultas e internamento de indivíduos adultos vivendo com HIV em Moçambique. Complicações associados a criptococose são praticamente inevitável na ausência de um diagnóstico e tratamento. A pesquisa irá implementar um diagnóstico laboratorial da criptococose usando amostra não invasiva (urina), com resultado disponível no intervalo de 15 minutos. Esta intervenção oferece a possibilidade de usar o teste de tinta de China em vez de simplesmente recorrer a tratamento empírico (quando o teste de rotina não está disponível).

A pesquisa vai apoiar no diagnóstico de indivíduos suspeitos de criptococose, contribuir para um início do tratamento mais próximo do momento da admissão.

**Objectivo da pesquisa**

Avaliar a implementação da técnica de tinta da China em urina como técnica de triagem laboratorial *point-of-care* da criptococose em pacientes adultos infectados por HIV no sul de Moçambique.

**Tipo de pesquisa/ Intervenção**

Trata-se de um estudo *stepped wedge trial* de implementação científica em que a intervenção será de forma faseada. A intervenção consiste no diagnóstico aprimorado da criptococose com a técnica da tinta-da-China usando a amostra não invasiva (urina).

**Selecção dos participantes**

Serão convidados a participar técnicos de laboratório do sector de microbiologia, clínicos afectos nos serviços de medicina dos seguintes hospitais: HCM, HGM, HGJM, HPM, HPX e HCC. A equipe do estudo irá pedir o consentimento informado por escrito a cada profissional de saúde elegível, incluindo autorização para gravar entrevista, fornecer dados profissionais, dados relacionados a Unidade sanitária/laboratório e procedimentos de cuidados e tratamento dos pacientes.

**Participação Voluntária**

Os participantes só serão envolvidos no estudo após terem lido ou escutado a leitura do Boletim informativo do estudo, terem percebido e concordarem em participar mediante a assinatura do consentimento informado. A participação é voluntária não é obrigado a participar da pesquisa e se você não concordar em participar a sua decisão não influencia no seu desempenho neste hospital. Caso decida participar e a dada altura queira interromper, poderá fazê-lo sem problema algum, bastando apenas comunicar a equipe do estudo, sem precisar de dizer os motivos para tal decisão.

**Procedimentos**

A equipe de estudo irá convidar os médicos e enfermeiros afectos ao serviços de medicina dos hospitais selecionados e técnicos de laboratório afectos ao sector de microbiologia a participar de forma voluntaria na pesquisa. Os que concordarem em participar serão convidados a assinar o consentimento informado e serão solicitados a participar de uma entrevista semi-estruturada de 25 minutos com gravação baseado num guião previamente criado e observação dos seus procedimentos técnicos. Na segunda fase do estudo serão solicitados a fazer parte de um treino relacionado com a implementação da técnica e na última fase serão solicitados a responder um questionário de perguntas fechadas relacionado com a avaliação da implementação.

Nesta pesquisa não se pretende avaliar o profissional de saúde ou repreende-lo, sendo assim ele deve se sentir não pressionado a dar respostas específicas nem se sintir envergonhado se não saber responder, mas pretendemos que respondesse com honestidade.

Portanto uma vez disponibilizado ao sistema nacional de saúde um recurso para o diagnóstico da criptococose será monitorado todo o processo de implementação tendo em conta a população de estudo os profissionais de saúde. A decisão do clinico solicitar amostra de urina para o teste da criptococose esta sobre a sua responsabilidade, não é obrigatório solicitar amostra de urina, de salientar que a participação dos clínicos no estudo é voluntária não é obrigatório. O que o estudo vai fazer é, fornecer ao sistema nacional de saúde um recurso para o diagnóstico da criptococose sendo que o uso desta tecnologia por parte dos clínicos é voluntária.

**Riscos, Desconfortos e Inconvenientes**

O estudo prevê o risco mínimo. Para mitigar o risco a base de dados não terá identificação dos profissionais e apenas os investigadores do estudo terão acesso a base de dados e será protegido por um código com acesso restrito. O estudo irá abordar os profissionais de saude em ambientes privados e fornecer uma linha de comunicação de acesso aos investigadores do estudo para apoiar, esclarecer as dúvidas e resolução das suas preocupações.

**Benefícios**

Os profissionais de saúde terão uma oportunidade de serem treinados para o diagnóstico da criptococose com uma técnica financeiramente de baixo custo, que usa uma amostra não invasiva (urina) de fácil obtenção, resultado no mesmo dia da consulta. Uma vez capacitados os profissionais de saúde terão possibilidade de recorrer a este teste para o rastreio laboratorial dos casos suspeitos de criptococose e tratem baseado no diagnóstico etiológico não apenas clinico (empírico).

**Custos da participação/Compensações**

Não haverá custo relacionado com a sua participação e não receberás nenhuma recompensa material nem monetária por participar do estudo.

**Confidencialidade e privacidade**

O estudo será realizado no seu hospital, no seu próprio consultório/laboratório. As entrevistas serão realizados no privado, numa sala identificada pelo estudo com acesso limitado. Serão usados códigos para anonimizar a sua participação. Ninguém que não seja investigador do estudo vai saber que esta a participar da pesquisa. Todos os esforços serão envidados para proteger a informação por si partilhada.

Todas informações recolhidas de profissionais de saúde serão muito bem guardadas e trancadas com cadeado no armário concebido para este estudo. As informações recolhidas serão usadas apenas responder os objectivos deste estudo e nunca para outras finalidades, não constara o nome do participante no relatório que será produzido.

**Partilha de Resultados**

Os resultados serão divulgados em forma de relatório, apresentações em conferências e publicações. O seu nome não constará nestes documentos.

**A quem contactar (Investigadores e Comité de Ética)**

Qualquer dúvida ou esclarecimento pode entrar em contacto com o Dr. José Langa pelo telefone: 84 7301265 ou 82 3885116.

Se não tiver obtido respostas satisfatórias, deverá entrar em contacto com o Comité Nacional de Bioética para a Saúde (CNBS) do Ministério da Saúde pelo telefone 824066350, que está situado na Av. Eduardo Mondlane/Salvador Allende - Maputo, 2^0^ andar.

## Versão 04, Outubro 2023

##

## CONSENTIMENTO INFORMADO

(PS)

Nome do participante: _________________________________________________

**Código do participante_________**

Tendo eu sido convidado a participar **no estudo:***“* **__________________________________”** Eu_____________________________________________declaro que:

1. Fui informado de forma satisfatória que a presente pesquisa tem por finalidade avaliar a implementação da técnica de tinta da China como técnica de triagem laboratorial *point-of-care* da criptococose em pacientes adultos infectados por HIV no sul de Moçambique;
2. Fui devidamente esclarecido da natureza da minha participação nesta pesquisa, dos riscos e benefícios que dela decorrem;
3. Compreendi que não receberei nenhuma recompensa material nem monetária por participar do estudo;
4. Fui devidamente esclarecido do direito que tenho em me retirar do estudo a qualquer momento sem qualquer prejuízo.
5. Compreendi que a informação relativa à minha participação terá carácter confidencial, e que a informação que irei partilhar será usada para responder os objectivos do presente estudo e igualmente para a tomada de decisão pelo Ministério de Saúde sobre futuros diagnósticos desta doença (criptococose) no hospital.
6. Compreendi que irei participar de uma entrevista de 25 minutos com gravação por áudio: ( ) sim ( ) não.
7. Compreendi também que se tiver perguntas as poderei fazer contactando a qualquer momento o investigador principal neste estudo, através do telefone número:+258 847301265.
8. Ou então se tiver alguma pergunta sobre os meus direitos em tanto que participante nesta pesquisa, ou se sentir que não foi tratado de forma adequada, poderei contactar o Comité Nacional de Bioética para a Saúde (CNBS) do Ministério da Saúde pelo telefone 824066350, que está situado na Av. Eduardo Mondlane/Salvador Allende - Maputo, 2^o^ andar.

| ____________________________________  Assinatura do participante  _____________________________________  Nome (em maiúsculas) do participante | _________________  Data e hora |
| --- | --- |

_________________________________________________ _________________

Assinatura da pessoa que realizou a explicação do consentimento Data e hora

| ____________________________________________  Nome (em maiúsculas) da pessoa que realizou a explicação do consentimento |  |
| --- | --- |

**Anexo I Versão 04, Outubro 2023**

**QUESTIONÁRIO**

(Clínicos e Técnicos de Laboratório)

**Implementação da técnica de tinta da China em urina para triagem laboratorial *point-of-care* da Criptococose em pacientes adultos infectados por HIV no sul de Moçambique.**

1. **INFORMAÇÃO DEMOGRÁFICA**

| **N^0^** | **Informação Demográfica** | **Resposta** |
| --- | --- | --- |
| 01 | Código do participante | ___ ___ ___/_______/_____/2023 |
| 02. | Unidade Sanitária: | 🞏HCM 🞏HGM 🞏HGJM 🞏 HPM 🞏HPX 🞏HCC |
| 03. | Idade | <30  ≥30  <40  ≥40 |
| 04. | Sexo | 🞏 Masculino 🞏Feminino |
| 05 | Nacionalidade | 🞏Moçambicana  🞏Estrageira |
| 06. | Qual é a sua principal área de trabalho ou unidade no hospital? (Por favor, marque UMA resposta) | 🞏 Medicina  🞏 Laboratório  🞏 Outra (por favor especifique:____________ |
| 07. | Qual é o seu nível de instrução? | 🞏 Médico internista  🞏 Residente em pós-graduação  🞏 Médico generalista  🞏 Enfermeiro Superior  🞏 Enfermeiro médio  🞏 Técnico de Laboratório  🞏 Outro (por favor especifique): ________ |
| 08. | Anos de prática | 🞏< 1 🞏 ≥1 🞏<5 🞏≥5 |

**Anexo II**  **Versão 04, Outubro 2023**

**QUESTIONÁRIO**

**Código ___ ___ ___/_______/_____/202___**

(RESPONSÁVEL DO LABORATÓRIO E TÉCNICOS**)**

**B/F**

**Implementação da técnica de tinta da China em urina para triagem laboratorial *point-of-care* da Criptococose em pacientes adultos infectados por HIV no sul de Moçambique.**

**ANÁLISE DA SITUAÇÃO ACTUAL**

| Código da US/LAB______ | Nível_____ | N^o^ de técnicos______ |
| --- | --- | --- |

1. **Com que frequência é efectuado o controlo de qualidade**

Sempre As vezes**** Nunca Não sei ****

1. **Indique o equipamento e material existente no seu laboratório**

Incubador  Cabine de biossegurança  Centrifuga  Microscópio  Lâminas microscópicas  Lamelas  Geleira 

1. **Qual é a condição da infraestrutura?**

Adequado  Inadequado 

1. **Com que frequência o laboratório tem material/equipamento para teste de criptococose?**

Sempre As vezes**** Nunca Não sei ****

1. **Com que frequência o laboratório tem reagentes para teste de criptococose?**

Sempre As vezes**** Nunca Não sei ****

1. **Estima o número de amostras para o diagnóstico da criptococose que são processadas por semana no seu laboratório**

<10 10 -- 20 ≥30

1. **Com que frequência em amostras suspeitos, é diagnosticado *Criptococcus* spp no seu laboratório?**

 100%  80 – 99% 50 – 79%  <50%

1. **Considera a criptococose uma doença negligenciada em Moçambique?**

Sim Não  Não sei 

1. **Seu laboratório sofre atrasos na emissão resultados ou interrompe o processamento das amostras devido à falta de reagentes?**

Sim  Não  Não sei 

1. **Você sente que a qualidade do trabalho é consistente em todos os turnos? (horário de trabalho, fim de semana, feriado, plantão noturno)**

Sim Não

1. **Se NÃO, na sua opinião em qual turno a qualidade do serviço do laboratório é comprometida?**

Horário de trabalho Fim de semana Feriado Plantão noturno

1. **Com que frequência você recebe treinamento em microscopia?**

1 treino/ano 2 treinos/ano nunca fui treinado

1. **Quando foi o seu último treino?**

>6 meses  ≥1 ano  ≥2 anos

1. **Indique por favor o seu nível de instrução**

Médico especialista  Médico generalista  Enfermeiro Técnico de Laboratório

1. **No laboratório existe base de dados eletrónico e funcional?**

Sim  Não  Não sei 

1. **Existe no laboratório livro de registo de dados?**

Sim  Não  Não sei ****

1. **O laboratório tem critérios para rejeição de amostras**

Sim  Não **** Não sei ****

1. **Que tipo de amostras são processadas no laboratório?**

Sangue Urina LCR Expetoração Biopsia

Outras (por favor especifique)_____________________________

1. **Com que frequência tem o EPI disponível (luvas) para manuseamento das amostras?**

Sempre As vezes Nunca Não sei 

1. **Que tipo de exames estão disponíveis no laboratório para diagnóstico de criptococose**

Tinta da China  CrAg  Cultura  Teste de aglutinação em latex (TAL) Ensaio imune enzimático (EIE) PCR

1. **Que microrganismos de origem fúngica são pesquisados no Laboratório**

*Criptococcus spp Candida spp Aspergillus spp Pneumocistes jirovecii*  Outros (por favor especifique)____________________

1. **O laboratório tem procedimentos operacionais padrão (POPs) de diagnóstico?**

Sim Não

1. **Qual é o tempo de resposta do Laboratório** (forneça sua melhor estimativa)

____min _____ horas

1. **Com que frequência são discutidos internamente os resultados dos pacientes antes de partilha com os clínicos?**

Sempre As vezes Nunca Não sei 

1. **Indique a forma de partilha de resultados com clínicos (**pode escolher mais do que uma resposta)

WhatsApp Email  Plataforma (*website*)  Agente de serviço faz chegar ao clínico Outra (por favor especifique)___________________

1. **Seu laboratório autoclava/incinera amostras, culturas ou material infecioso antes de descartar?**

Sim Não Não sei

1. **Fornecimento de água corrente no seu laboratório**

<12h/dia 12 - 16h/dia 24h/dia

1. **Fornecimento de energia no seu laboratório**

<12h/dia 12 - 16h/dia 24h/dia

1. **Tem alternativa de fornecimento de energia (gerador)?**

Sim Não

1. **Que outros desafios/dificuldades você encontra para efectuar um exame de *Cryptococcus?***

**Obrigado pela colaboração.**

**Anexo III**  **Versão 04, Outubro 2023**

**GUIÃO DE ENTREVISTA AO CLÍNICO**

**Código ___ ___ ___/_______/_____/202___**

**Implementação da técnica de tinta da China em urina para triagem laboratorial *point-of-care* da Criptococose em pacientes adultos infectados por HIV no sul de Moçambique.**

**DIAGNÓSTICO CLÍNICO-LABORATORIAL E *FEEDBACK* DO LAB**

1. Encontra muitos casos de criptococose enquanto cuidas de pacientes no seu consultório?
2. Você se sente habilitado o suficiente para cuidar de pacientes com doença criptocócica?
3. Você se sente a vontade para cuidar de pacientes com doença criptocócica?
4. Que sinais e sintomas seus pacientes com doença criptocócica costumam apresentar?
5. Quais doenças imitam ou se parecem com a doença criptocócica no seu consultório?
6. Que tipo de teste habitualmente solicita quando suspeita uma doença criptocócica?
7. Na sua opinião o pessoal do laboratório está disponível quando você tenta contatá-los?
8. A punção lombar está sempre disponível na sua US?
9. Que desafios você encontra se tiver que referir pacientes para punção lombar com o outro médico?
10. Em que se apoia para o diagnóstico da criptococose na sua Unidade Sanitária?
11. Tem alguns critérios para decidir por um tratamento seja sindrómico em vez do direcionado?
12. Considera complexo ou simples o fluxo de atendimento incluindo diagnóstico laboratorial e tratamento?
13. O que constitui barreira no fluxo de atendimento, diagnóstico laboratorial e tratamento de pacientes suspeito da criptococose?
14. Na sua opinião, que aspectos podem facilitar a aderência ao novo teste de diagnóstico laboratorial da criptococose na sua US/LAB
15. Tendo em conta as dificuldades que motivações encontras no âmbito de atendimento e tratamento de pacientes suspeitos de criptococose?
16. Em que frequência as suas decisões terapêuticas foram suportados por Laboratório?
17. Estima o tempo de resposta Laboratorial para disponibilidade do resultado.

**Obrigado pela colaboração.**

**Anexo IV.**  **Versão 03, Setembro 2023**

**QUESTIONÁRIO**

**Código ___ ___ ___/_______/_____/202___**

**Implementação da técnica de tinta da China em urina para triagem laboratorial point-of-care da Criptococose em pacientes adultos infectados por HIV no sul de Moçambique**

**(Práticas/conduta _Clínico)**

1. **Indique o principal método de diagnóstico de criptococoses em uso no seu laboratório**

Clinico ; Tinta da China ; CrAg ; Cultura ; Teste de aglutinação em latex (TAL) ; Ensaio imune enzimático (EIE); PCR

1. **Com que frequência solicitas o teste?**

Sempre As vezes**** Nunca Não sei ****

1. **Com que frequência o teste de *Criptococcus* esta disponível?**

Sempre As vezes**** Nunca Não sei ****

1. **Tempo de resposta do Laboratório** (forneça sua melhor estimativa)

______min _______horas

1. **Tempo de início de tratamento após o resultado positivo** (forneça sua melhor estimativa)

______horas ______dias

1. **Considera a criptococose uma doença negligenciada em Moçambique?**

Sim Não 

1. **Antes de solicitar o teste que factores considera? (**pode escolher mais do que uma resposta)

**** Gravidade da doença

 Carga do trabalho na clínica/laboratório

 Idade do Paciente

 Disponibilidade de serviços de laboratório

CD4<100cell/mm^3^

CD4<100cell/mm^3^

 Outro (Faz favor mencionar):____________________________________________

1. **Qual é a sua opinião sobre o formulário de solicitação dos enxames laboratoriais?**

Adequado  Inadequado 

1. **O laboratório notifica as enfermarias quando há introdução de um novo exame incluindo os seus parâmetros?**

Sim Não

1. **O laboratório notifica imediatamente os resultados críticos**

Sim Não

1. **Com que frequência o laboratório avisa a você quando há interrupção de um determinado exame?**

Sempre As vezes Nunca Não sei 

1. **Você sente que a qualidade do trabalho é consistente em todos os turnos? (horário de trabalho, fim de semana, feriado, plantão noturno)**

Sim Não

1. **Se NÃO, na sua opinião em qual turno a qualidade do serviço é comprometida?**

Horário de trabalho Fim de semana Feriado Plantão noturno

1. **Quantos pacientes assiste por semana? (forneça sua melhor estimativa)**

<10 10 - 20 ≥30

1. **Qual é o perfil do paciente que chega á US**

Crítico moderado

1. **Assinale os sinais/sintomas da criptococose que observas nos seus pacientes**

cefaleia febre rigidez da nuca convulsões acne

Outro (por favor mencione):__________________________

1. **Existem critérios para triagem?**

Sim  Não 

1. **Guião de definição de caso disponível**

Sim  Não 

1. **O rastreio de criptococose em pacientes HIV faz parte da rotina na sua US?**

Sim  Não 

1. **O que constitui barreira para o diagnóstico e tratamento de pacientes suspeito da criptococose? (**pode escolher mais do que uma resposta)

 Baixa auto-confiança motivado por sobrecarga do trabalho no Laboratório

 Rotura de reagentes

 Rotura do teste

 Dificuldades para obtenção da amostra

Outro (Faz favor mencionar):_____________________________________________

1. **Com que frequência você confia completamente os resultados do teste do paciente**

 100%  80 – 99% 50 – 79%  <50%

1. **Quais factores podem fazer com que você trate criptococose na presença de um resultado** **negativo do teste?**

 Forte suspeita clínica

 O resultado do teste pode ser indeterminado, incorrecto ou inconsistente

 Idade do paciente

 Preferência

 Outro (Faz favor mencionar):____________________________________________

1. **O pessoal do laboratório está disponível quando você tenta contatá-los?**

Sim  Não 

1. **Na sua opinião o laboratório disponibiliza a segunda via do resultado quando você solicita?**

Sim  Não **** Nem sempre

1. **Anfotericina B esta geralmente disponível na Unidade sanitária?**

Sim  Não ** **Não sei Nem sempre

1. **Os pacientes podem adquirir fluconazol gratuitamente na Unidade sanitária?**

Sim  Não ** **Não sei

1. **Há registo de seguimento dos pacientes com resultados positivos para Criptococose**

 Sim

Não, os pacientes não retornam depois da primeira visita

1. **No livro tem informações detalhado de contacto dos pacientes positivos para *Criptococcus*?**

Sim  Não **** Não sei 

1. **Se for solicitado para melhorar algo no diagnóstico e tratamento da criptococose que alterações sugeria?**

**Obrigado pela colaboração.**

**Anexo V**  **Versão 04, Outubro 2023**

**Implementação da técnica de tinta da China em urina para triagem laboratorial *point-of-care* da Criptococose em pacientes adultos infectados por HIV no sul de Moçambique.**

**QUESTIONÁRIO**

**Código ___ ___ ___/_______/_____/202___**

**AVALIAÇÃO DA INTERVENÇÃO**

**(Clinico)**

1. **As ferramentas disponibilizadas no treino são mais esclarecedores no âmbito de diagnóstico e tratamento da criptococose**

Concordo fortemente Concordo Neutro Descordo Descordo fortemente

1. **A intervenção é uma alternativa recomendável para triagem laboratorial da Criptococose**

Concordo fortemente Concordo Neutro Descordo Descordo fortemente

1. **Desde a sua implementação o teste da tinta-da-China a partir da urina esteve sempre disponível no Hospital**

Concordo fortemente Concordo Neutro Descordo Descordo fortemente

1. **Qualidade dos serviços melhorou com o novo diagnóstico laboratorial**

Concordo fortemente Concordo Neutro Descordo Descordo fortemente

1. **Pode-se estimar a percentagem de pacientes beneficiados pela intervenção em:**

 100%  80 – 99% 50 – 79%  <50%

1. **Avalie a sua satisfação em relação ao diagnostico laboratorial com a técnica da tinta da China usando a urina**

Muito satisfeito Satisfeito Neutro insatisfeito Muito insatisfeito

1. **Avalie a sua satisfação em relação a disponibilidade do pessoal do laboratório quando você tenta contatá-los?**

Muito satisfeito Satisfeito Neutro insatisfeito Muito insatisfeito

1. **Avalie a sua satisfação em relação ao tempo de resposta do laboratório**

Muito satisfeito Satisfeito Neutro insatisfeito Muito insatisfeito

1. **Avalie a sua satisfação em relação ao formulário de solicitação dos enxames laboratoriais**

Muito satisfeito Satisfeito Neutro insatisfeito Muito insatisfeito

1. **Avalie a sua satisfação em relação a clareza, legibilidade e integridade do resultado do laboratório relatório?**

Muito satisfeito Satisfeito Neutro insatisfeito Muito insatisfeito

1. **A qualidade do diagnóstico foi consistente em todos os turnos? (horário de trabalho, fim de semana, feriado, plantão noturno)**

Concordo fortemente Concordo Neutro Descordo Descordo fortemente

1. **Na sua opinião em qual turno, a qualidade do serviço do laboratório foi comprometida?**

Horário de trabalho Fim de semana Feriado Plantão noturno NA

1. **O número de pacientes internados devido a complicações associadas a meningite criptocócica reduziu com a introdução da nova abordagem do diagnóstico**

Concordo fortemente Concordo Neutro Descordo Descordo fortemente

1. **Com a introdução da nova abordagem do diagnóstico o número de óbitos reduziu**

Concordo fortemente Concordo Neutro Descordo Descordo fortemente

1. **A nova abordagem do diagnóstico contribuiu para que maior número de indivíduos vivendo com HIV tivessem oportunidade de testar para *Criptococcus***

Concordo fortemente Concordo Neutro Descordo Descordo fortemente

1. **O tratamento de pacientes com meningite criptocócica melhorou com implementação da nova abordagem para diagnóstico laboratorial**

Concordo fortemente Concordo Neutro Descordo Descordo fortemente

1. **Avalie na generalidade a sua satisfação em relação a intervenção no seu hospital**

Muito satisfeito Satisfeito Neutro insatisfeito Muito insatisfeito

**Obrigado pela colaboração.**

**Anexo V**  **Versão 04, Outubro 2023**

**Implementação da técnica de tinta da China em urina para triagem laboratorial *point-of-care* da Criptococose em pacientes adultos infectados por HIV no sul de Moçambique.**

**QUESTIONÁRIO**

**Código ___ ___ ___/_______/_____/202___**

**AVALIAÇÃO DA INTERVENÇÃO**

**(Técnico de Laboratório)**

1. **Avalie o seu nível de confiança ao efectuar o exame de tinta de China usando urina**

****Muito confiante Confiante Neutro Inseguro Muito inseguro

1. **As ferramentas disponibilizadas no treino são mais esclarecedores no âmbito de diagnóstico e tratamento da criptococose**

Concordo fortemente Concordo Neutro Descordo Descordo fortemente

1. **A intervenção é uma alternativa recomendável para triagem laboratorial da Criptococose**

Concordo fortemente Concordo Neutro Descordo Descordo fortemente

1. **Desde a sua implementação o teste da tinta-da-China a partir da urina esteve sempre disponível no Hospital**

Concordo fortemente Concordo Neutro Descordo Descordo fortemente

1. **Qualidade dos serviços melhorou com o novo diagnóstico laboratorial**

Concordo fortemente Concordo Neutro Descordo Descordo fortemente

1. **Pode-se estimar a percentagem de pacientes beneficiados pela intervenção em:**

 100%  80 – 99% 50 – 79%  <50%

1. **Avalie a sua satisfação em relação ao diagnostico laboratorial com a técnica da tinta da China usando a urina**

Muito satisfeito Satisfeito Neutro insatisfeito Muito insatisfeito

1. **Avalie a sua satisfação em relação a clareza e legibilidade dos exames solicitados pelo clínico?**

Muito satisfeito Satisfeito Neutro insatisfeito Muito insatisfeito

1. **Avalie a sua satisfação em relação a disponibilidade dos clínicos quando você tenta contatá-los?**

Muito satisfeito Satisfeito Neutro insatisfeito Muito insatisfeito

1. **Avalie a sua satisfação em relação ao tempo de resposta do laboratório**

Muito satisfeito Satisfeito Neutro insatisfeito Muito insatisfeito

1. **Avalie a sua satisfação em relação ao formulário de solicitação dos enxames laboratoriais**

Muito satisfeito Satisfeito Neutro insatisfeito Muito insatisfeito

1. **A qualidade do diagnóstico foi consistente em todos os turnos? (horário de trabalho, fim de semana, feriado, plantão noturno)**

Concordo fortemente Concordo Neutro Descordo Descordo fortemente

1. **Na sua opinião em qual turno, a qualidade do serviço do laboratório foi comprometida?**

Horário de trabalho Fim de semana Feriado Plantão noturno NA

1. **Com a introdução da nova abordagem do diagnóstico o número de óbitos reduziu**

Concordo fortemente Concordo Neutro Descordo Descordo fortemente

1. **A nova abordagem do diagnóstico contribuiu para que maior número de indivíduos vivendo com HIV tivessem oportunidade de testar para *Criptococcus***

Concordo fortemente Concordo Neutro Descordo Descordo fortemente

1. **Avalie na generalidade a sua satisfação em relação a intervenção no seu hospital**

Muito satisfeito Satisfeito Neutro insatisfeito Muito insatisfeito

**Obrigado pela colaboração.**
