# Supplementary material for: Re-introduction of India ink testing as a low-cost laboratory diagnostic for cryptococcosis among HIV infected patients in Southern Mozambique: An implementation research protocol
Source: PLoS One. 2025 May 23;20(5):e0324792. doi: 10.1371/journal.pone.0324792 (PMC12101839; doi:10.1371/journal.pone.0324792)
Supplement: S3 File — (DOCX) [file pone.0324792.s003.docx]

**DOCTORATE IN BIOSCIENCES AND PUBLIC HEALTH**

Research Project Title

**Implementation of the India ink technique in urine for point-of-care laboratory screening of Cryptococcosis in adult HIV-infected patients in southern Mozambique**

Version 04, October 2023

Maputo, 11/2023

**DOCTORATE IN BIOSCIENCES AND PUBLIC HEALTH**

Research Project Title

**Implementation of the India ink technique in urine for point-of-care laboratory screening of Cryptococcosis in adult HIV-infected patients in southern Mozambique**

Student name: José Carlos Langa, Faculty of Medicine, UEM

Supervisors:

Main Supervisor: Professor Mohsin Sidat, School of Medicine, UEM

Co-Supervisor: Professor Jahit Sacarlal, School of Medicine UEM

Co-Supervisor: Professor Troy D. Moon, Tulane University School of Public Health and Tropical Medicine-USA

Versão 04, Outubro 2023

Maputo, 11/2023

**Contents**

[1. Motivation 7](#_Toc189476107)

[2. Objective 9](#_Toc189476108)

[3. Contribution 9](#_Toc189476109)

[4. Problem 9](#_Toc189476110)

[4.1 Research Questions 11](#_Toc189476111)

[5. Literature Review 10](#_Toc189476114)

[6. Conceptual framework 13](#_Toc189476115)

[7. Methods 15](#_Toc189476116)

[7.1 Study design and type 15](#_Toc189476117)

[7.2 Study Site 18](#_Toc189476118)

[7.3 Study Timeline 19](#_Toc189476119)

[7.4 Study population 17](#_Toc189476120)

[7.5 Sample and Sampling 17](#_Toc189476121)

[7.6. Inclusion and exclusion criteria 18](#_Toc189476123)

[7.9. Studies 20](#_Toc189476124)

[7.9. Variables and data analysis plan 25](#_Toc189476128)

[8. Study limitation 27](#_Toc189476129)

[9. Ethical considerations 27](#_Toc189476130)

[9.1. Potential risks and how these will be minimized 28](#_Toc189476131)

[9.2. Informed consent 28](#_Toc189476132)

[9.3. Confidentiality 28](#_Toc189476133)

[9.4. Potential benefits 29](#_Toc189476134)

[9.6. Expected results 29](#_Toc189476135)

[9.7. Dissemination of results 30](#_Toc189476136)

[10. Study Timeline 31](#_Toc189476137)

[11. Study Team 32](#_Toc189476138)

[11.1. Role of each team member 32](#_Toc189476139)

[12. Budget 33](#_Toc189476144)

[13. References 34](#_Toc189476145)

[14. APPENDICES AND ANNEXES 37](#_Toc189476146)

**Abbreviations and acronyms**

AIDS-Acquired Immunodeficiency Syndrome

ART- Antiretroviral treatment T

CDA- Complete Diagnostic Autopsy

CDC- Center for Disease Control and Prevention

CHC- Carmelo Hospital of Chokwé

CM-Cryptococcal meningitis

CNBS- National Bioethics Committee for Health

CNS-Central Nervous System

CrAg- Cryptococcal antigen

CSF- Cerebrospinal fluid Cerebrospinal fluid

DAP-Dynamic Adaptation Process

HIV - Human Immunodeficiency Virus

HP-Health professional

ID - Patient identification

IFI - Invasive Fungal Infections

JMGM- José Macamo General Hospital

MCH- Maputo Central Hospital

MGH- Mavalane General Hospital

MoH -Ministry of Health

NHS-National Health System

OR-“Odds Ratio”

RDT- Rapid diagnostic test

UEM-Universidade Eduardo Mondlane

USA- United States of America

US-Unidade Sanitária

VD-Dependent variable

WHO-World Health Organization

**Abstract**

In the world, cryptococcal meningitis (CM) associated with HIV infection has been identified as the cause responsible for around 150,000 - 200,000 deaths per year, occurring mainly in the sub-Saharan African region, with a record of over 70%; 135,900 [95% CI 93,900-163,900]. Limitations in the diagnosis and treatment of patients with HIV-associated CM may be the probable cause of the high mortality rate in the sub-Saharan African region.

The World Health Organisation (WHO) recommends screening and treatment of cryptococcosis in all HIV patients in order to avoid complications and reduce the mortality rate. However, in countries with limited resources, there is a need to implement diagnostics that are appropriate to the country's reality, simple and inexpensive in order to avoid missing diagnostic opportunities due to rapid test breakdowns. The India ink technique in urine has been indicated as an alternative for the diagnosis of Cryptococcus, as urine can easily detect the presence of this pathogen, especially in individuals with disseminated infection.

The main objective of the study is to implement the India ink technique in urine for point-of-care laboratory screening of cryptococcosis in adult HIV-infected patients in southern Mozambique.

A stepped wedge trial of scientific implementation with a mixed approach will be carried out. Sampling will be non-probabilistic by convenience and intentional. Data collection will be done through questionnaires, observation, and semi-structured interviews with health professionals (clinicians and laboratory technicians). Implementation will be in phases and a Dynamic Adaptation Process (DAP) conceptual framework will be used then the intervention will be evaluated using the RE-AIM (Reach, Effectiveness, Adoption, Implementation, and Maintenance) conceptual framework.

It is hoped that the findings of this study will provide advocacy so that the Ministry of Health (MoH) can prioritize diagnostic strategies with low-cost non-invasive samples and maximize the diagnosis, control, and prevention of CM which, together with HIV, are devastating the country's health and socio-economic resources.

# Motivation

During the coordination of surveillance of invasive fungal infections (IFI) at Maputo Central Hospital, there were challenges related to the diagnosis and breakdown of the rapid tests currently in use, which is a limitation for the timely diagnosis of cryptococcosis. In addition, diagnosis using an invasive CSF sample has been a barrier in places with few health professionals with the skills to collect the sample. The India ink technique in urine is simple and less expensive and has been indicated as an alternative for diagnosing Cryptococcus.

IFIs have recently taken center stage among the diseases that affect the health and well-being of the population worldwide. With the advent of HIV/AIDS, IFI is one of the main causes of consultation, hospitalization, and death, especially in sub-Saharan Africa. Studies show that prolonged treatment with antibacterials without a cure is probably caused by fungi. Mortality from cryptococcosis is practically inevitable in the absence of proper diagnosis and treatment. Gaps in care and diagnosis can contribute to poor management of fungal infections, especially in Mozambique where they are neglected.

According to data from INSIDA 2021, the prevalence of HIV in the adult population in Mozambique is 12.5%(1). HIV is a risk factor for IFIs, and in Mozambique, the most complex IFI diagnosis is carried out at the Central Hospitals and academic reference laboratories of the Department of Microbiology at the Faculty of Medicine of Universidade Eduardo Mondlane (UEM). The priority is to implement a diagnosis of cryptococcosis that is appropriate to the country's reality, simple and inexpensive, in order to avoid missed diagnostic opportunities due to the breakdown of rapid tests and to improve the management of IFIs in the context of care and treatment, which has been little done.

The introduction of timely non-invasive diagnosis, its promotion, and its expansion to cover a greater proportion of HIV patients suspected of having cryptococcosis can contribute to starting treatment closer to the time of admission, improve patient outcomes, and contribute to reducing mortality.

# Objectives

2.1. **Main objective**

- Evaluate the implementation of the India ink technique in urine as a point-of-care laboratory screening technique for cryptococcosis in adult HIV-infected patients in southern Mozambique.
- 2.2. Specific objectives
- To analyze the existing practices and challenges in the laboratory diagnosis of cryptococcosis in the following hospitals MCH, MGH, JMJH, MPH, XPH, and CHC;
- Implement the technique of laboratory diagnosis of cryptococcosis using the India ink method in urine as a point of care in the selected hospitals;
- To evaluate the reach, efficacy, adoption, implementation, and maintenance of the India ink method in urine as a point-of-care laboratory diagnosis for cryptococcosis.

# Contribution

Providing acceptable healthcare in sub-Saharan Africa is a complex process, due to the scarcity of human and material resources and extremely limited access to diagnostic tests.

Each year, approximately 12 million people die in sub-Saharan Africa (2,3) and most of these deaths are not investigated. The few deaths that are investigated are generally attributed to infectious diseases (4), most commonly HIV infection, malaria and tuberculosis.

In the absence of laboratory confirmation, the accuracy of these estimates remains uncertain.

In Mozambique, due to the growing number of people at risk of cryptococcosis combined with various factors including HIV, the introduction of timely, less expensive cryptococcosis diagnosis with a non-invasive sample (urine) and a result on the same day as the consultation, could contribute to an increase in laboratory confirmation of suspected cryptococcosis cases and guide clinicians with an aetiological diagnosis to start treatment. It could be expanded to the country's peripheral hospitals, contribute to diagnosis and treatment in patients where invasive samples are not accessible, and allow treatment to be started as soon as the patient is admitted.

The implementation is also intended to be sustainable, used for continuous (routine) screening, and to alleviate socio-economic resources in the country.

# Problem

Sub-Saharan Africa has the highest burden of invasive/opportunistic infections associated with HIV/AIDS (5). Cryptococcosis is an important opportunistic infection and one of the main causes of consultation and hospitalization in adults living with HIV in sub-Saharan Africa (6).

In Mozambique, approximately 2,097,000 (two million and ninety-seven thousand) adults are living with HIV, with a prevalence of 12.5%, according to data from INSIDA 2021(1).

The classic diagnosis of cryptococcosis is carried out by testing for the microorganism in cerebrospinal fluid (CSF) or blood. Invasive samples are currently being replaced by alternative non-invasive samples that are easy to obtain and have shown the same performance without harming the patient. Studies show that there are gaps in the diagnosis of fungal infections associated with non-comprehensive diagnosis of cryptococcosis, limited access to rapid diagnostic tests (RDTs), especially in developing countries, and that little has been done to improve progress in diagnosis (7,8).

In Mozambique, IFI is neglected and data is very scarce. Few published studies estimate the annual incidence of cryptococcal meningitis (CM) at 18,600 cases (70.5 cases per 100,000 people/year), and the country ranks third among the countries with the highest incidence of CM (8,9). The recently published study using complete diagnostic autopsy (CDA) in 284 deceased patients from Mozambique (n=223) and Brazil (n=61) showed that Cryptococcus was responsible for 16 deaths among the 163 HIV-positive patients (10%; 95 % CI: 6-15%), including four maternal deaths (6).

More accurate non-invasive diagnosis at the time of admission can improve patient outcome and contribute to reducing mortality from cryptococcosis.

## Research questions

# What opportunities exist to implement the point-of-care diagnostic technique for cryptococcosis in the context of care and treatment of cryptococcosis in HIV patients?

# What factors contribute to the reach, efficacy, adoption, implementation, and maintenance of India ink as a point-of-care diagnostic technique for cryptococcosis in the selected hospitals?

# Literature Review

Worldwide, data published up to 2021 showed that HIV-associated CM was responsible for 150,000-200,000 deaths a year(10). These deaths occurred mainly in sub-Saharan Africa, where mortality was over 70%; 135,900 [95% CI 93,900-163,900] (7,9,10,11). One of the main reasons for this high mortality is the delay in diagnosis, associated with limited access to lumbar puncture and diagnostic tests(13). In this region, studies show that despite efforts to provide antiretroviral treatment (ART), there is an increase in the burden of opportunistic invasive infections, including cryptococcosis (13,14).

Most HIV-associated infections are caused by *Cryptococcus neoformans*, although in Botswana, up to 30% of infections are caused by *Cryptococcus gattii(*11).

CM is among the most underfunded diseases in the world. It has recently been proposed as a neglected tropical disease (15). It is believed that prolonged treatment with antibacterial agents and failure to cure HIV patients with headaches and fever is probably caused by Cryptococcus spp (16,17). Death from CM is practically inevitable in the absence of adequate treatment (5).

The prevalence of specific fungal pathogens depends on their endemicity. Recently published studies show that 70% of all cryptococcosis cases in some African countries appear to support HIV diagnosis and approximately 30% after the start of ART (18,19).

Early identification of cryptococcal infection is extremely crucial (20). Screening, early treatment and intervention have been shown to be viable for reducing the course of the disease and deaths from CM in HIV/AIDS patients (14). In sub-Saharan Africa, providing acceptable healthcare has been a major challenge due to the scarcity of resources and extremely limited diagnostic tests (7).

The diagnosis of cryptococcosis has been carried out in the past using a sample of cerebrospinal fluid (CSF) with India ink and isolation of the yeast in Sabouraud dextrose culture medium. However, urine is currently a highly desirable sample for the diagnosis of cryptococcosis in places with limited resources, few doctors, and other health specialists mainly in primary-level hospitals (18,21), particularly when there are difficulties in obtaining invasive samples such as blood or CSF. Cryptococcus spp in urine is an indicator of disseminated cryptococcosis(22). In the majority of cases in the country, CSF is collected by specialized clinicians.

Urine samples have the potential to increase the chance of early diagnosis of cryptococcosis in many settings, as they are easy to obtain (18,23).

Studies have shown agreement of results (sensitivity and specificity) using invasive samples (blood and CSF) when compared to non-invasive samples (urine) in the diagnosis of cryptococcosis (18,24). Saha et al. (24) in their findings found a positive predictive value ranging from (82.9, to 100%) for china ink using urine samples, which suggests a good test for screening. This diagnostic method has a sensitivity of (85-86.1%) and a specificity of 100%, and the result is obtained on the same day as the sample is taken.

The WHO (25) and other studies published by Greene et al., 2020 and Stott et al., 2021 recommend the use of cryptococcal antigen (CrAg) testing for cryptococcosis screening in patients living with HIV. However, some studies show the unsustainability of CrAg in relation to costs in low-income countries when compared to India ink and also public hospitals show more confidence in the most commonly used diagnostic means for cryptococcosis, India ink and culture (26,27).

Cryptococcosis is an opportunistic mycosis that is transmitted by inhaling viable propagules of Cryptococcus spp. The fungus is found in the environment, in pigeon droppings, eucalyptus and decaying organic matter (13,28). The yeast has two recognised varieties Cryptococcus neoformans var. neoformans (serotypes A, D and AD), which causes over 90% of infections, and Cryptococcus neoformans var. gattii (serotypes B and C). The grubii variant has recently been described and is related to serotype A. The fungus has a sexual or teleomorphic phase which consists of the formation of basidiospores (29). In the parasitic phase, i.e. in the tissues, the microorganism appears as a capsulated yeast and sometimes with budding. When inhaled, the fungus first reaches the lungs where it can be latent in alveolar macrophages or proliferate with tropism for the central nervous system (CNS), causing CM (28,29).

Currently, cryptococcosis has gained greater prominence and its incidence has increased with the emergence of HIV. The increase in the incidence of IFIs is associated with the growing number of patients with risk factors for these infections. Other conditions and associated risk factors are: neutropenia, diabetes mellitus and kidney infection, intensive care hospitalization, use of immunomodulators, extensive surgery or burns, and invasive interventions (12).

Patients with cryptococcosis present with headache and fever with an average duration of two weeks between the onset of symptoms and the first signs. Many patients develop nausea, vomiting, diplopia due to cranial nerve paralysis (CN VI), and reduced visual acuity related to high CSF pressure. If left untreated, symptoms progress to an abnormal mental state, reduced level of consciousness, seizures, and finally coma (11).

Clinically significant invasive disease is associated with the reactivation of latent infection among immunocompromised individuals, such as people living with HIV, months to years after initial exposure (13).

With regard to treatment, according to the World Health Organisation (WHO) and the Centre for Disease Control and Prevention (CDC), the antifungal intravenous amphotericin B and oral fluconazole have been shown to be effective for the treatment of cryptococcosis (25,30). The WHO recommends treating asymptomatic cryptococcal infection with oral fluconazole and close monitoring in order to reduce possible side effects (25).

# Conceptual framework

This study will be guided by the conceptual framework called the Dynamic Adaptation Process (DAP) that will support the implementation of the non-invasive diagnostic method for cryptococcosis in the selected hospitals and another called RE-AIM with five domains (Reach, Effectiveness, Adoption, Implementation and Maintenance) to evaluate the implementation (Figure 2). The PAD involves identifying essential elements and adaptable features (barriers and facilitators) based on evidence, supporting implementation with specific training on adaptations allowed to the model, monitoring fidelity, and supporting and identifying the need and solutions for adapting workflow in the context of cryptococcosis care and treatment (31,32).

Fidelity refers to an assessment of the adherence and competence of health professionals. Adherence to the diagnosis of cryptococcosis with non-invasive sampling that will be implemented in this study meets the expectations of the model's creators and will be measured as one of the intervention's indicators. The ability of health professionals to use the model is called competence. The PAD model has four implementation phases (pre-implementation, adaptation, implementation and evaluation), takes into account the multi-level context of service delivery, involves various stakeholders and provides knowledge and continuous feedback. During the intervention, it guides and provides ‘steps’ for monitoring the implementation of the diagnosis.


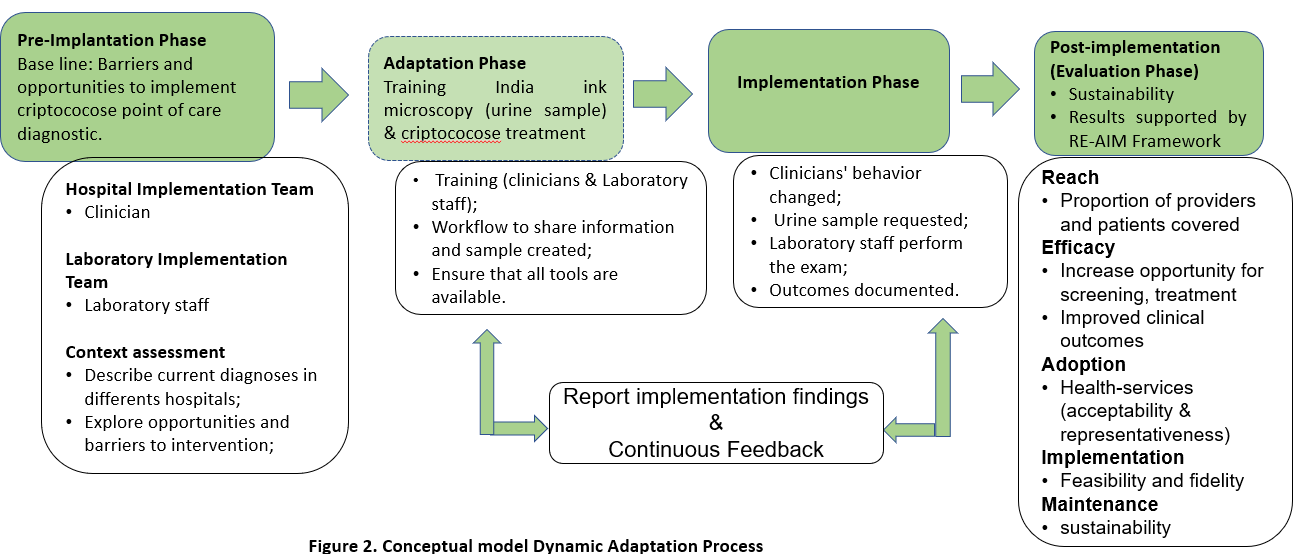


# Methods

## Study design and type

The study design is a stepped wedge trial with a mixed approach. Stepped wedge studies are prospective and intervention studies. The main characteristic of these studies is that the researcher sets up the implementation in phases, monitors the conditions of the intervention, and makes it possible to assess the effectiveness of the intervention tool or technique implemented. This type of study ensures that all selected study sites eventually receive the intervention and generally provides more reliable evidence (See Table 1).

**Tabela 1. Desenho de estudo *Stepped-wedge***

| Phases | Months/2024-2025 | | | | | | | | | | | | | | |
| --- | --- | --- | --- | --- | --- | --- | --- | --- | --- | --- | --- | --- | --- | --- | --- |
|  | 1 | 2 | 3 | 4 | 5 | 6 | 7 | 8 | 9 | 10 | 11 | 12 | 1 | 2 | 3 |
| MCH | C | C | C | C | PI | AD | I | FU | FU | FU | FU | FU | FU | FU | FU |
| MGH | C | C | C | C | PI | AD |  | I | FU | FU | FU | FU | FU | FU | FU |
| JMGH | C | C | C | C | C | PI | AD |  | I | FU | FU | FU | FU | FU | FU |
| MPH | C | C | C | C | C | PI | AD |  |  | I | FU | FU | FU | FU | FU |
| XPH | C | C | C | C | C | C | PI | AD |  |  | I | FU | FU | FU | FU |
| CHC | C | C | C | C | C | C | PI | AD |  |  |  | I | FU | FU | FU |

**C-** Control**; PI**-Pré-implementation; **AD**- Adaptation; **I**-Implementation; **FU**- follow-up

The mixed method approach will allow triangulation and complementarity of the findings, giving the possibility of strengthening the research conclusions (33). The quantitative part will be cross-sectional and the qualitative part will be generic. Quantitative data such as (the number of tests carried out with Chinese ink, positive results, negative results, response time, level of academic education of health professionals, number of health professionals allocated to HIV services, number of trained professionals, percentage of services that adhered to the intervention, number of microscopes in existence) will be obtained through a questionnaire administered to health professionals and by observation based on the previously drawn up script.

In the qualitative part, through semi-structured interviews, topics will be explored that address (challenges for: diagnosis, and treatment of cryptococcosis, and the satisfaction of health professionals in relation to the intervention will also be evaluated).

## Study site

The study will be carried out in three provinces in southern Mozambique (Maputo City, Maputo Province and Gaza Province), where the following hospitals will be selected for convenience: Maputo Central Hospital (MCH), Mavalane General Hospital (MGH), José Macamo General Hospital (JMGH), Matola Provincial Hospital (MPH), Xai-Xai Provincial Hospital (XPH) and Carmel Hospital of Chokwé (CHC). These are the most distinguished hospitals in the southern part of the country.

**Description of each study site:**

MCH is a reference hospital in the country, located in the city of Maputo, in the Central neighbourhood. It is situated between Eduardo Mondlane, Salvador Allende, Tomás Nduda and Agostinho Neto Avenues. It is a quaternary level hospital and the most differentiated in the country, with University Hospital status; MGH is a tertiary level hospital and is located in the city of Maputo, KaMavota Urban District, Mavalane neighbourhood, on Avenida das Forças Populares de Libertação de Moçambique (FPLM) number 798; JMGH is a tertiary level hospital and is located on Avenida da Organização da Unidade Africana (OUA), Nlhamankulu Urban District in Maputo; HPM is a tertiary level hospital and is located in Matola City, Maputo Province; HPX is a tertiary level hospital and is located in Xai-Xai City, Gaza Province; HCC is a tertiary level hospital located in the district of Chokwé, is a reference centre for Tuberculosis and AIDS patients in Gaza Province, and is managed and administered by the Sisters of Charity of St Vincent de Paul, by agreement with the MoH.

In terms of capacity, the hospitals selected for the study operate with a capacity of approximately (239-1500) official beds and have the following clinical services: medicine, orthopaedics, paediatrics, surgery, gynaecology-obstetrics, oncology, emergency and resuscitation services. They are NHS hospitals and are managed by the MoH. It should be noted that Hospital Carmelo, a referral hospital for tuberculosis and AIDS patients, is managed and administered by the Sisters of Charity of St Vincent de Paul, in agreement with the MoH. The hospitals were selected for convenience (existence of a research partnership agreement for many years, they have HIV and inpatient services).

## Study timeline

- The study will last approximately 1 year and 3 months with different phases:
- First phase: Assessment of the current situation: (duration 3 months):
- Second phase: Adaptation (duration 3 months);
- Third phase: Implementation of the intervention in phases guided by the PAD (duration 6 months);
- Fourth phase: Evaluation, sustainability and results according to the RE-AIM (duration 3 months).

## Study population

- Doctors, nurses, and laboratory technicians from the following hospitals: MCH, MGH, JMGH, MPH, XPH and CHC.

## Sample and Sampling

**Sample for the qualitative component of the study**

The sample size will be determined based on two principles: previous studies of the implementation of techniques in hospitals and theoretical saturation. The sample in previous studies ranges from ‘24-30 health professionals’. Since Mozambique has a shortage of health professionals, it was determined that all available professionals (doctors, nurses and laboratory technicians) working in the medicine and microbiology laboratories of the selected hospitals would be involved in the study.Profissionais por serem envolvidos

MCH: 8 clinicians from Medicine and 2 laboratory technicians;

MGH: 4 clinicians from Medicine and 2 laboratory technicians;

HGJM: 4 clinicians from Medicine and 2 laboratory technicians;

MPH: 3 clinicians from Medicine and 2 laboratory technicians;

XPH: 3 clinicians from Medicine and 2 laboratory technicians;

CHC: 2 clinicians from Medicine and 2 laboratory technicians.

.

**Sampling**

Sampling will be non-probabilistic, with participants selected intentionally and study sites (hospitals) selected for convenience. The involvement of the selected hospitals is due to the existence of a research partnership agreement with them for many years, and they also have medical services associated with ART and hospitalisation, which is a great advantage for the implementation of this study.

The researcher will have no contact with the patients and will not recruit them. The clinician will decide which patients to ask for urine samples to diagnose cryptococcosis. The study will provide the national health system with a resource for diagnosing cryptococcosis.Descrição da técnica a ser implementada

## The India ink in urine technique is a quantitative, low-cost, easy-to-perform and easy-to-maintain technique. It is carried out using a non-invasive sample (urine) that is easy to obtain. A drop of urine of approximately 2ml is deposited on the microscope slide, and then a drop of China ink is added and covered with the coverslip. The result is obtained in approximately 15 minutes and allows capsulated yeasts suggestive of Cryptococcus spp. to be observed.

## 7.6. Inclusion and exclusion criteria

**7.6.1. Inclusion criteria**

- Laboratory technicians assigned to the Microbiology sector, doctors and nurses assigned to the medical services of each selected hospital will be included.

**7.6.2. exclusion criteria**

- All health professionals who are not directly involved in the microbiological diagnosis and care of adult HIV patients will be excluded.

**7.8. Procedures, instruments for collecting and analysing data**

Quantitative data will be collected by the researcher based on the observation guide and questionnaire attached to the REDCap platform loaded onto the tablet. Demographic information will be collected from the professionals, and all the equipment and materials currently in use for the care and treatment of cryptococcosis will be listed and described, including the collection of information related to the infrastructure of each health unit.

It will also describe the cryptococcosis diagnoses currently carried out in each hospital involved in the study and identify barriers and facilitators to the implementation of the cryptococcosis diagnosis technique using urine samples.

Based on the DAP conceptual framework, laboratory diagnosis of cryptococcosis will be implemented in the six selected hospitals, training will be carried out, a flow of information will be created between laboratory technicians and clinicians, the definition of a cryptococcosis case will be standardized and diagnostic and treatment guidelines will be provided.

During implementation, clinicians will request urine samples from eligible patients and will be performed by trained laboratory technicians, monitoring will be carried out, the intervention will be documented and continuous feedback between clinicians and laboratory technicians will be ensured.

The implementation and results will be evaluated based on the RE-AIM conceptual framework of five domains: reach (comprehensiveness), efficacy, adoption (acceptability and representativeness), implementation and maintenance (sustainability) of the intervention in hospitals.

**Analyzing - Qualitative data**

The researcher will collect qualitative data through semi-structured interviews administered to health professionals, lasting 25 minutes each, and recorded on a digital recorder.

The analysis of qualitative data will be based on Nvivo software version 12. A thematic analysis will be carried out, with room for the emergence of hypotheses and new theories in relation to the topic.

The recorded interviews will be transcribed in Word without altering their originality and classified into previously created categories. Participants will be anonymized using numbers and the initials of their names. Quality control of the transcripts will be carried out by comparing what has been written with the audio of the interview to ensure that no information has been omitted or added, thus guaranteeing the originality of the information.

NVivo software will be used to help organize and control the data and the study will also be supported by the DAP and RE-AIM conceptual frameworks. The text passages will be grouped into themes according to the conceptual frameworks.

**This qualitative component will generate the following constructs:**

i. Health professionals' knowledge of the burden of cryptococcosis in their hospital and in the country;

ii. Health professionals' knowledge of cryptococcosis diagnosis methods in their hospital;

iii. Perceived barriers to screening, diagnosis, and treatment of cryptococcosis in your hospital;

iv. Survey: Opportunities for the new diagnosis of cryptococcosis in your hospital.

**Quantitative data analysis**

Quantitative data will be analyzed using the R studio statistical package. Univariate descriptive analyses will be carried out (mean or median age, standard deviation and variance will be calculated), percentage frequencies of the following variables: tests requested, doctors/laboratory technicians who adhered to the intervention, chi-square to check for associations between the intervention/test and the variables (level of education of the professional, availability of the test, severity of the disease and gender) and logistic regression to estimate the relationship between adherence to the test and factors severity of the disease, work overload in the US/laboratory, level of education of the professional, shift, gender, availability of laboratory services.

A Likert scale will be used to assess the level of satisfaction of all those involved in the intervention (doctors and laboratory technicians) during the study period. And the frequencies of the information obtained will be calculated.

The results will be described and presented in tables and graphs using the Microsoft Excel program. A p-value of <0.05 will be considered significant.

## 7.9. Studies

The study includes three sub-studies:

# Practices and challenges in the laboratory diagnosis of cryptococcosis in six hospitals in the three southern provinces of Mozambique.

This is a cross-sectional study with a mixed approach. The qualitative approach will be generic, where the current situation will be assessed before the intervention is implemented (Figure 2: Pre-implementation phase) in order to identify elements of the social, health, administrative, and structural context that may or may not favor the intervention in the local context.

The assessment of the current situation will be carried out in six hospitals (MCH, MGH, JMGH, MPH, XPH, and CHC) over three months. Sampling will be non-probabilistic, and intentional with regard to the participants and by convenience with regard to the study sites.

The data for this study will be obtained through semi-structured questionnaires (Annexes I, II, and IV) and interviews (Annex III) with health professionals from each hospital (clinicians from the medical service and microbiology laboratory technicians); and a list will be made of the equipment, existing material and a description of the cryptococcosis diagnoses currently carried out in each hospital involved in the study and to identify barriers and facilitators to implementing the technique of diagnosing cryptococcosis with urine samples.

The data will be analyzed using the R studio statistical package, where the following analyses will be carried out: descriptive analysis (calculating frequencies, mean or median, standard deviation, and variance); and chi-square to compare frequencies. The results will be described and presented in tables and graphs. A p-value of <0.05 will be considered significant.

# To implement the technique of laboratory diagnosis of cryptococcosis using the China ink method in urine as a point of care in selected hospitals in three southern provinces of Mozambique.

This is a stepped wedge trial. Sampling will be non-probabilistic by convenience in relation to the study sites and purposive in relation to the participants.

The study will be carried out in six hospitals (MCH, MGH, JMGH, MPH, XPH, and CHC) over six months. The Adaptation Phase will be the beginning of the iteration with the new test, the healthcare professionals (clinicians in medical service and laboratory technicians assigned to the microbiology sector) adapt to the workflow associated with the intervention (Figure 2). During the three months of adaptation, the researcher will carry out training on the care, diagnosis and treatment of cryptococcosis using urine dye (5 to 20 ml of the sample) with intentionally selected healthcare professionals, the diagnostic guide tools will be provided and an information-sharing workflow will be established between the laboratory and the clinicians.

It's up to the clinician to decide which patients to ask for a urine sample to diagnose cryptococcosis and also to decide what clinical information to share with the laboratory according to each situation. We will provide the national health system with a resource for the diagnosis of cryptococcosis, instead of empirical treatment when routine treatment is not available. Therefore, the clinician is free to request routine or experimental testing. If the clinician decides to request a urine sample for the diagnosis of cryptococcosis, the laboratory will provide feedback according to the clinician's request in order to follow up on the patient.

The implementation of the diagnosis of cryptococcosis with Chinese ink using non-invasive urine samples at the selected sites will take place over 6 months. In this implementation phase, the health professionals assigned to the medical service with knowledge of the intervention (new test for cryptococcosis) will request that it be carried out by colleagues from the laboratory who have been previously trained in its execution. Throughout this phase (Figure 2) monitoring of clinicians' behaviour in relation to the use of the new diagnostic will take place and documentation of its implementation will be captured (Appendix V).

The data will be analysed using the R studio statistical package. The frequencies of the variables (number of tests requested with and without feedback, number of trained professionals) will be calculated and the frequencies will be compared using the Chi-square test.

The correlation between (number of professionals trained and number of tests requested) will be determined; the Odds Ratio (OR) will be estimated using regression: services covered/improved (dependent variable) and independent variables: professional trained, years of practice, level of education of the professional; age, gender.

# Evaluation of the RE-AIM-based implementation of the technique of laboratory diagnosis of cryptococcosis using the China ink method in urine as a point of care in southern Mozambique

This is a cross-sectional quantitative study aimed at measuring the contribution of the diagnosis introduced (intervention effect) in the six hospitals (MCH, MGH, JMGH, MPH, XPH and CHC) over three months. Sampling will be non-probabilistic, intentional with regard to the participants (healthcare professionals) and by convenience concerning the study sites.

In the Implementation Evaluation Phase (Post-implementation), during three months the implementation will be evaluated using the RE-AIM conceptual framework with five domains reach (comprehensiveness), effectiveness, adoption (acceptability and representativeness), implementation and maintenance (sustainability) of the intervention in the hospitals. Data collection will be based on previously prepared questionnaires and standardized scripts coupled to the REDCap platform loaded onto the tablet.

- Reach will be calculated through the proportion of individuals covered by the intervention (clinicians and laboratory technicians).
- Efficacy will be assessed by checking laboratory and clinical results, time to diagnosis and satisfaction of health professionals.

The study includes an evaluation at the beginning (baseline) pre-implementation and at the end post-implementation, where laboratory and clinical results, time to diagnosis and satisfaction of health professionals will be verified at both times. The efficacy of the intervention will therefore be evaluated before and after implementation. WHO recommendations will be consulted, which recommend screening and treatment to prevent complications from a cryptococcal infection. The test to be implemented will contribute to starting treatment on the same day as the consultation and based on the aetiological diagnosis.

- Adoption will be explored through the representativeness of the services that have adopted the implementation including sample flow, laboratory processing and feedback, use of scripts drawn up and made available during the intervention.
- A Likert scale will also be used to analyze the satisfaction of all health professionals covered by the intervention during the study period (clinicians and laboratory technicians).
- During implementation, the fidelity of health professionals in relation to the diagnosis, care and treatment of cryptococcosis based on documented observation will be assessed.
- Maintenance (sustainability) will be assessed based on the simplicity of the intervention (laboratory diagnostic technique for cryptococcosis using urine), the workflow and the cost of the test implemented.

The data obtained in this study will be entered into the R studio statistical package, where the following analyses will be performed: Descriptive analysis (calculate frequencies, mean and variance), Chi-square to compare frequencies and multivariate regression to estimate the “Odds Ratio” (OR).

## Variables and data analysis plan

| **Objective** | **Variables** | **Data Analysis Plan** |
| --- | --- | --- |
| To analyze existing practices and challenges in the laboratory diagnosis of cryptococcosis in the hospitals MCH, MGH, JMGH, MPH, XPH, and CHC. | Hospital level, education level, nationality, years of practice, gender, age, marital status, types of tests requested, availability of tests, number of samples tested, participation in quality control, microscope availability, centrifuge availability, availability of the reagent, workflow per shift. | Descriptive analysis calculates the frequency of (tests requested, samples tested, type of test, sex, years of practice, age) and mean, standard deviation, variance; Chi-square to establish a relationship between the level of education, gender and the type of test requested  Independent t-test: relationship between workflow per shift and test type, number of tests requested with and without feedback.  Qualitative data, Nvivo version 12: content analysis |
| To Implement the laboratory diagnostic technique for Cryptococcosis using the Indian ink method in urine as a point of care in selected hospitals. | Hospital level, healthcare professional's level of education, trained professional, number of tests requested, type of tests requested, number of samples tested, response time, use of provided scripts, completeness of data filled in. | Frequency: test requests, lab results shared by technicians, Chi-square (relationship between test request and lab feedback); Correlation: (Number of trained professionals and number of tests requested); Multivariate regression to estimate the “Odds Ratio” (OR) of the relationship between the request for VD swarm & independent variables: professional education level, years of practice, hospital level, shift, work overload in the clinic/laboratory, age of the professional, sex, use of provided scripts. |
| To evaluate the implementation of the India ink method as a point of care laboratory diagnosis in selected hospitals, based on the RE-AIM conceptual framework. | Number of services and number of providers that adhered to implementation, age, sex, number of samples tested, test availability, initiation of treatment after positive test. | Frequency (professionals who adhered to the intervention, samples tested, services that adhered to the intervention, age and sex. Chi-square compare frequencies Ex: (relationship between characteristics of professionals and the type of test requested);  Multivariate regression to estimate the “Odds Ratio” (OR) of the relationship between adherence to testing (PS) and the variables: severity of the disease, age of the professional, sex, US, availability of services in the laboratory, and feedback |

# Study limitation

Some limitations were identified, namely:

- The nature of the stepped wedge study, which is long and involves following up participants, poses a risk of losing participants, introducing bias into the analysis of outcomes;

To minimize this fact, all clinicians from the medical service and laboratory technicians from the microbiology sector will be involved in the training and cross-sectional analyses of the outcomes will be carried out.

- Data collection through self-administered questionnaires is likely to be a limitation because participants may respond to please the institution and its managers, and using interviews to collect qualitative data risks participants responding in a way they think is appropriate. the researcher wants to hear;

To minimize this fact, the questionnaires will be anonymous, the researcher will be present when the questionnaire is administered and during the interviews to clarify the interviewee regarding the questions.

# Ethical considerations

The data collected for this study will not contain information that allows the identification of the participants involved, they will be coded and anonymized. Participation will be subject to signing the informed consent form; the link between the code and the participants' names will only be known by the researchers. Confidentiality will be maintained at all times during the research, the data will only be used for this study and never for other purposes.

The study will not change the type of cryptococcosis diagnostic sample in force in the hospitals involved. The study will demonstrate to clinicians and laboratory technicians an alternative for diagnosing cryptococcosis using a non-invasive and easy-to-obtain sample. Patients diagnosed with cryptococcosis will be treated in accordance with current Ministry of Health (MoH) regulations.

The researchers declare that they will comply with good research ethics practices. The study will be submitted to the Boards of Directors of the selected Hospitals and to the Institutional Committee for Bioethics in Health of the Faculty of Medicine/Maputo Central Hospital (CIBS FM & HCM). It will also be reviewed and approved by the National Committee on Bioethics for Health (CNBS).

## Potential risks and how these will be minimized

The study foresees minimal risks for the participants involved in the study, such as discomfort, fatigue, and anxiety. To mitigate risk during training, the importance of ensuring and strengthening information sharing between clinicians and laboratory technicians will be emphasized, and a line of communication will also be provided for study researchers to clarify their doubts, provide support, and resolve issues. your concerns.

## Informed consent

The informed consent process begins with pre-study inclusion, when the study team explains all pertinent information related to the study. Study personnel will explain to each potential participant the nature of the study, its purpose, the procedures involved, the expected duration, the potential risks and benefits involved, and any discomfort that may be associated with participation in the study. See informed consent in the appendix on page 38.

## Confidentiality

The confidentiality of the information collected is extremely fundamental. Research staff will be trained to adhere to, comply with, and follow standards strictly and protect participant confidentiality.

The following measures will be used to protect the confidentiality of the information provided:

- During training, the importance of maintaining confidentiality will be emphasized to the research team;
- Interviews will be conducted in private settings;
- Signed informed consent forms will be filed in a locked file cabinet separately from other study data;
- Participants’ contact details will be stored securely on password-protected computers separately from other study data;
- There will be no registration of names in any data collection terms, therefore, we will use unique identification numbers (IDs) that will be assigned to each participant;
- Unintentionally collected information that is identified in the recordings will be edited;
- Data will be entered directly into a password protected device.
- Data will be regularly uploaded to a secure server.
- Only a limited number of study personnel whose roles require access to identifying information (i.e., signed informed consent form, contact information, master list relating participant names to study ID number) will have access to this type of data;
- Participants will not be identified by their own names in any reports or publications resulting from the research data.

Information will be collected through the REDCap (Research Electronic Data Capture) platform loaded onto the tablet. All copies of data, electronic files including Microsoft Word transcripts and databases will be securely archived at the School of Medicine for a period of five years after the completion of the research..

## Potential benefits

Health professionals will have an opportunity to be trained in the diagnosis of cryptococcosis with a cost-effective technique that uses a non-invasive sample (urine) that is easily obtained. Once trained, healthcare professionals will be able to use this test for laboratory screening of suspected cases of cryptococcosis and treat them based on the etiological diagnosis, not just the clinical (empirical) one.

- 1. Declaration of conflicts of interest

The researchers declare that there is no conflict of interest in the present study, nor monetary benefits resulting from conducting the study.

- 1. Expected results

It is expected that:

- The study provides evidence of the practices and challenges existing in the laboratory diagnosis of Cryptococcosis;
- Health professionals are trained, diagnostic opportunities are increased and treatment is initiated closer to the time of admission to improve patient outcomes;
- The Indian ink technique for point-of-care laboratory screening is implemented and functional;
- The data can contribute information to MoH and provide opportunities for advocacy so that less expensive cryptococcosis diagnostic strategies are prioritized, maximizing the control and prevention of this neglected disease that, together with HIV, is devastating health and resources. socioeconomic in the country.
  1. Dissemination of results

The results will be presented first at the data collection sites (hospitals), then in the PhD program, at scientific conferences and finally published in scientific journals.

# Study Timeline

| **ACTIVITIES** | **MONTHS /2022 – 2026** | | | | | | | | | | | |
| --- | --- | --- | --- | --- | --- | --- | --- | --- | --- | --- | --- | --- |
|  | **02/2022** | **03-07/2022** | **08-12/2022** | **01-03/2023** | **04-12/2023** | **01/2024-03/2024** | **04/2024-12/2024** | **01-06/2025** | **07-09/2025** | **10/2025-11/2025** | **12/2025-03/2026** | **04/2026** |
| Literature review |  |  |  |  |  |  |  |  |  |  |  |  |
| Protocol preparation |  |  |  |  |  |  |  |  |  |  |  |  |
| Project Submission to the Doctoral Program |  |  |  |  |  |  |  |  |  |  |  |  |
| Defense of the research project |  |  |  |  |  |  |  |  |  |  |  |  |
| Submission of the Project to the Institutional Committee of Bioethics in Health of FM & MCH and CNBS |  |  |  |  |  |  |  |  |  |  |  |  |
| Assessment of the current situation (Pre-implementation phase) |  |  |  |  |  |  |  |  |  |  |  |  |
| Adaptation and implementation phase of the intervention |  |  |  |  |  |  |  |  |  |  |  |  |
| Manuscript Submission I |  |  |  |  |  |  |  |  |  |  |  |  |
| English and Ethics |  |  |  |  |  |  |  |  |  |  |  |  |
| Implementation Evaluation Phase and Manuscript Submission II |  |  |  |  |  |  |  |  |  |  |  |  |
| Manuscript Submission III |  |  |  |  |  |  |  |  |  |  |  |  |
| Specialty course and preparation of the financing proposal |  |  |  |  |  |  |  |  |  |  |  |  |
| Writing sabbatical |  |  |  |  |  |  |  |  |  |  |  |  |
| Thesis defense |  |  |  |  |  |  |  |  |  |  |  |  |

# Study Team

José Langa, Resident of the Doctoral program in Biosciences and Public Health at the Faculty of Medicine, UEM, Principal Investigator;

Professor Mohsin Sidat, Faculty of Medicine, UEM, Supervisor;

Professor Troy D. Moon, Tulane School of Public Health and Tropical Medicine-USA, Supervisor;

Professor Jahit Sacarlal, Faculty of Medicine, UEM_Supervisor.

Focal point (Clinician and laboratory technician) – MCH;

Focal point (Clinician and laboratory technician) – MGH;

Focal point (Clinician and laboratory technician) – JMGH;

Focal point (Clinician and laboratory technician) – MPH;

Focal point (Clinician and laboratory technician) – XPH;

Focal point (Clinician and laboratory technician) – CHC.

- 1. Role of each team member
- Principal Investigator: Facilitate training, data collection, analysis, and report writing;
- Clinical focal points for each US: Data management in the US and sharing with the principal investigator;
- Laboratory technicians: sample processing
- Supervisors/Mentors: Guide the researcher in conducting the research, writing the report, and identifying opportunities for disseminating the research results.

# Budget

| **Item** | **Amount** | **Unit cost (Mt)** | **Total (Mt)** |
| --- | --- | --- | --- |
| **Office Supplies** | | | |
| Pencil | 40 | 20,00 | 800,00 |
| Rubber | 40 | 20,00 | 800,00 |
| Pencil spinner | 40 | 20,00 | 800,00 |
| Pen | 40 | 25,00 | 1.000,00 |
| Ream of paper | 25 | 400,00 | 10.000,00 |
| Print |  |  | 30.000,00 |
| Copy |  |  | 22.000,00 |
| Recorder | 1 |  |  |
| **Subtotal 1** | | | **65.400,00** |
|  | | | |
| **Laboratory Material** | | | |
| India ink (30ml) | 6kits | 400,00 | 1.600,00 |
| Microscopic slides | 12cx | 975,00 | 11.700,00 |
| Lamellae | 12cx | 1410,00 | 16.920,00 |
| Gloves |  |  | 20.550,00 |
| **Subtotal 2** | | | **50.770,00** |
|  | | | |
| **Support Staff** | | | |
| Health professionals | 6 | 20.000,00 | 120.000,00 |
| **Subtotal 3** | | | **120.000,00** |
|  | | | |
| **Travel and communication** | | | |
| Travel and communication | | | 80.000,00 |
| **Subtotal 4** | | | **80.000,00** |
|  | | | |
| **Total** | | | **316.170,00** |
| **Unforeseen 10%** | | | **31.617.00** |
| **Total with 10% of unforeseen events** | | | **347.787.00** |

# References

1. INSIDA. INQUÉRITO NACIONAL SOBRE O IMPACTO DO HIV E SIDA EM MOÇAMBIQUE INSIDA 2021.

2. World Health Organization (WHO). The Health of the people: The African Regional health report. 2006.

3. UNICEF. Monitoring and statistics. In 2005.

4. World Health Organization (WHO). The world health report 2004—changing history. Geneva. 2004.

5. French N, Gray K, Watera C, Nakiyingi J, Lugada E, Moore M, et al. Cryptococcal infection in a cohort of HIV-1-infected Ugandan adults: AIDS. 2002 May;16(7):1031–8.

6. Hurtado JC, Castillo P, Fernandes F, Navarro M, Lovane L, Casas I, et al. Mortality due to Cryptococcus neoformans and Cryptococcus gattii in low-income settings: an autopsy study. Sci Rep. 2019 Dec;9(1):7493.

7. Petti CA, Polage CR, Quinn TC, Ronald AR, Sande MA. Laboratory Medicine in Africa: A Barrier to Effective Health Care. Clinical Infectious Diseases. 2006 Feb 1;42(3):377–82.

8. Rajasingham R, Smith RM, Park BJ, Jarvis JN, Govender NP, Chiller TM, et al. Global burden of disease of HIV-associated cryptococcal meningitis: an updated analysis. The Lancet Infectious Diseases. 2017 Aug;17(8):873–81.

9. Sacarlal J, Denning D. Estimated Burden of Serious Fungal Infections in Mozambique. JoF. 2018 Jun 23;4(3):75.

10. Stott KE, Loyse A, Jarvis JN, Alufandika M, Harrison TS, Mwandumba HC, et al. Cryptococcal meningoencephalitis: time for action. The Lancet Infectious Diseases. 2021 Sep;21(9):e259–71.

11. Limper AH, Adenis A, Le T, Harrison TS. Fungal infections in HIV/AIDS. The Lancet Infectious Diseases. 2017 Nov;17(11):e334–43.

12. Jarvis JN, Bicanic T, Loyse A, Namarika D, Jackson A, Nussbaum JC, et al. Determinants of Mortality in a Combined Cohort of 501 Patients With HIV-Associated Cryptococcal Meningitis: Implications for Improving Outcomes. Clinical Infectious Diseases. 2014 Mar 1;58(5):736–45.

13. WHO. Guidelines for the diagnosis, prevention and management of cryptococcal disease in HIV-Infected adults, adolestents and childrens. 2018.

14. Greene G, Lawrence DS, Jordan A, Chiller T, Jarvis JN. Cryptococcal meningitis: a review of cryptococcal antigen screening programs in Africa. Expert Review of Anti-infective Therapy. 2021 Feb 1;19(2):233–44.

15. Molloy SF, Chiller T, Greene GS, Burry J, Govender NP, Kanyama C, et al. Cryptococcal meningitis: A neglected NTD? Zunt JR, editor. PLoS Negl Trop Dis. 2017 Jun 29;11(6):e0005575.

16. Ordi J, Castillo P, Garcia-Basteiro AL, Moraleda C, Fernandes F, Quintó L, et al. Clinico-pathological discrepancies in the diagnosis of causes of death in adults in Mozambique: A retrospective observational study. Moreira J, editor. PLoS ONE. 2019 Sep 6;14(9):e0220657.

17. Bongomin F, Gago S, Oladele R, Denning D. Global and Multi-National Prevalence of Fungal Diseases—Estimate Precision. JoF. 2017 Oct 18;3(4):57.

18. Jarvis JN, Percival A, Bauman S, Pelfrey J, Meintjes G, Williams GN, et al. Evaluation of a Novel Point-of-Care Cryptococcal Antigen Test on Serum, Plasma, and Urine From Patients With HIV-Associated Cryptococcal Meningitis. Clinical Infectious Diseases. 2011 Nov 15;53(10):1019–23.

19. Jarvis JN, Meintjes G, Harrison TS. Outcomes of cryptococcal meningitis in antiretroviral naive and experienced patients in South Africa. J Infect. 2010;496–8.

20. Chastain DB, Henao-Martínez AF, Franco-Paredes C. Opportunistic Invasive Mycoses in AIDS: Cryptococcosis, Histoplasmosis, Coccidiodomycosis, and Talaromycosis. Curr Infect Dis Rep. 2017 Oct;19(10):36.

21. Sara. Invetario Nacional. 2018.

22. Kiertiburanakul S, Sungkanuparph S, Buabut B, Pracharktam R. Cryptococcuria as a Manifestation of Disseminated Cryptococcosis and Isolated Urinary Tract Infection.

23. Pinto Junior VL, Galhardo MCG, Lazéra M, Wanke B, Reis RS, Perez M. Criptococose associada à Aids: a importância do cultivo da urina no seu diagnóstico. Rev Soc Bras Med Trop. 2006 Apr;39(2):230–2.

24. Saha DC, Xess I, Biswas A, Bhowmik DM, Padma MV. Detection of Cryptococcus by conventional, serological and molecular methods. Journal of Medical Microbiology. 2009 Aug 1;58(8):1098–105.

25. World Health Organization. Rapid advice Diagnosis, Prevention anD ManageMent of CryPtoCoCCal Disease in Hiv-infeCteD aDults, aDolesCents anD CHilDren. 2011.

26. Deiss R, Loreti CV, Gutierrez AG, Filipe E, Tatia M, Issufo S, et al. High burden of cryptococcal antigenemia and meningitis among patients presenting at an emergency department in Maputo, Mozambique. Kufa T, editor. PLoS ONE. 2021 Apr 26;16(4):e0250195.

27. Rajasingham R, Wake RM, Beyene T, Katende A, Letang E, Boulware DR. Cryptococcal Meningitis Diagnostics and Screening in the Era of Point-of-Care Laboratory Testing. Kraft CS, editor. J Clin Microbiol [Internet]. 2019 Jan [cited 2021 Aug 6];57(1). Available from: https://journals.asm.org/doi/10.1128/JCM.01238-18

28. Murray PR Ken and Pfaller, Michael. Microbiologia Medica. 6th ed. 2010.

29. Jawetz, Melnick, & Adelberg’s. Medical Microbiology. 26th Edition. 2013.

30. Centers for Disease Control and Prevention (CDC). Cryptococcal Screening Program Training Manual for Healthcare Providers. 2012.

31. Aarons GA, Green AE, Palinkas LA, Self-Brown S, Whitaker DJ, Lutzker JR, et al. Dynamic adaptation process to implement an evidence-based child maltreatment intervention. Implementation Sci. 2012 Dec;7(1):32.

32. Glasgow RE, Vogt TM, Boles SM. Evaluating the public health impact of health promotion interventions: the RE-AIM framework. Am J Public Health. 1999 Sep;89(9):1322–7.

33. Schoonenboom J, Johnson RB. How to Construct a Mixed Methods Research Design. Köln Z Soziol. 2017 Oct;69(S2):107–31.

34. Naing L, Winn T, Rusli BN. Practical Issues in Calculating the Sample Size for Prevalence Studies. :6.

# APPENDICES AND ANNEXES

**Participant Information Sheet and Informed Consent Form**

**(PS)**

**PART I**

**Protocol title**

**Implementation of the Indian ink technique in urine for point-of-care laboratory screening of Cryptococcosis in HIV-infected adult patients in southern Mozambique**

Protocol version: 04, October 2023

Consent Form/Participant Information Sheet Version: 04, October 2023

Principal researcher: José Langa^1^;

Co-Investigators: Mohsin Sidat^1^ ; Jahit Sacarlal1 and Troy D. Moon^2^

**Researchers Affiliation**

1. Faculty of Medicine - Eduardo Mondlane University

2. Tulane School of Public Health and Tropical Medicine-USA

**Name of Funder/Sponsor**

Partnership for Research in Implementation Science – Mozambique (PRISM) a collaboration between Eduardo Mondlane University (UEM) and Tulane University School of Public Health and Tropical Medicine-USA with funding from Fogarty International Center of the National Institutes of Health of the United States of America ( USA).

NB: Please always ask a study team member to explain anything you do not understand while reading this informed consent form and do not sign until all your questions are answered.

**Introduction:**

Cryptococcosis is an opportunistic infection and one of the main causes of consultation and hospitalization of adults living with HIV in sub-Saharan Africa. Early diagnosis can prevent complications from this infection.

The classic diagnosis of cryptococcosis is made by searching for the microorganism in the cerebrospinal fluid (CSF) or blood. Currently, invasive samples are replaced by alternative non-invasive samples, which are easy to obtain and do not harm the patient.

This study will be conducted at the following hospitals: Maputo Central Hospital, Mavalane General Hospital, José Macamo General Hospital, Matola Provincial Hospital, Xai-Xai Provincial Hospital, and Carmelo Hospital of Chokwé.

The Informed Consent Form sets out the reason for the research. It explains the procedures, possible risks, and benefits if you agree to participate in the study.

**Research justification**

Cryptococcosis has recently taken on a prominent role among the diseases that affect the health and well-being of the population around the world. Cryptococcosis is a neglected opportunistic disease associated with an increase in the number of consultations and hospitalizations of adults living with HIV in Mozambique. Complications associated with cryptococcosis are virtually inevitable in the absence of proper diagnosis and treatment. The research will implement a laboratory diagnosis of cryptococcosis using a non-invasive sample (urine), with results available within 15 minutes. This intervention offers the possibility of using the India ink test instead of simply resorting to empirical treatment (when routine testing is not available).

The research will support the diagnosis of individuals suspected of cryptococcosis, contributing to the start of treatment closer to the time of admission.

**Research objective**

To evaluate the implementation of the Indian ink technique in urine as a point-of-care laboratory screening technique for cryptococcosis in HIV-infected adult patients in southern Mozambique.

**Type of research/Intervention**

This is a stepped wedge trial scientific implementation study in which the intervention will be phased. The intervention consists of the improved diagnosis of cryptococcosis with the Indian ink technique using the non-invasive sample (urine).

**Selection of participants**

Laboratory technicians from the microbiology sector and clinicians allocated to the medical services of the following hospitals will be invited to participate: MCH, MGH, JMGH, MPH, XPH, and CHC. The study team will ask each eligible healthcare professional for written informed consent, including permission to record interviews, provide professional data, data related to the health facility/laboratory, and patient care and treatment procedures.

**Voluntary Participation**

Participants will only be involved in the study after they have read or listened to the study newsletter, understood it, and agreed to participate by signing the informed consent form. Participation is voluntary, you are not obliged to participate in the research and if you do not agree to participate, your decision will not influence your performance in this hospital. If you decide to participate and at any time want to stop, you can do so without any problem, simply by informing the study team, without having to give reasons for your decision.

**Procedures**

The study team will invite doctors and nurses working in the medical services of the selected hospitals and laboratory technicians working in the microbiology sector to voluntarily participate in the research. Those who agree to participate will be asked to sign the informed consent and will be asked to participate in a 25-minute semi-structured interview with recording based on a previously created script and observation of their technical procedures. In the second phase of the study they will be asked to take part in training related to the implementation of the technique and in the last phase they will be asked to answer a closed-ended questionnaire related to the evaluation of the implementation.

This research does not intend to evaluate the health professional or reprimand him/her, so he/she should not feel pressured to give specific answers nor should he/she feel embarrassed if he/she does not know how to answer, but we expect him/her to answer honestly.

Therefore, once a resource for diagnosing cryptococcosis is made available to the national health system, the entire implementation process will be monitored, taking into account the study population and health professionals. The clinician's decision to request a urine sample for the cryptococcosis test is his/her responsibility. It is not mandatory to request a urine sample. It is worth noting that the clinician's participation in the study is voluntary and not mandatory. What the study will do is provide the national health system with a resource for diagnosing cryptococcosis, with the use of this technology by clinicians being voluntary.

**Risks, Discomforts, and Inconveniences**

The study predicts minimal risk. To mitigate the risk, the database will not identify the professionals and only the study researchers will have access to the database and it will be protected by a code with restricted access. The study will target healthcare professionals in private settings and provide a line of communication to access study investigators to support, clarify doubts, and address concerns.

**Benefits**

Health professionals will have the opportunity to be trained to diagnose cryptococcosis using a low-cost technique that uses a non-invasive sample (urine) that is easy to obtain, with results on the same day of the consultation. Once trained, healthcare professionals will be able to use this test for laboratory screening of suspected cases of cryptococcosis and treat them based on the etiological diagnosis, not just the clinical (empirical) one.

**Participation Costs/Compensations**

There will be no cost associated with your participation and you will not receive any material or monetary reward for participating in the study.

**Confidentiality and privacy**

The study will be carried out in your hospital, in your own office/laboratory. The interviews will be conducted in private, in a room identified by the study with limited access. Codes will be used to anonymize your participation. No one who is not a researcher in the study will know that you are participating in the research. Every effort will be made to protect the information shared by you.

All information collected from healthcare professionals will be carefully stored and locked in a cabinet designed for this study. The information collected will only be used to meet the objectives of this study and never for other purposes. The participant's name will not appear in the report that will be produced.

**Dissemination Results**

The results will be published in the form of a report, conference presentations, and publications. Your name will not appear on these documents.

**Who to contact (Investigators and Ethics Committee)**

For any questions or clarifications, please contact Dr. José Langa by phone: 84 7301265 or 82 3885116.

If you have not received satisfactory answers, you should contact the National Committee for Bioethics for Health (CNBS) of the Ministry of Health by calling 824066350, located at Av. Eduardo Mondlane/Salvador Allende - Maputo, 20th floor.

## Version 04, October 2023

##

**INFORMED CONSENT**

**(PS)**

Participant Name: _________________________________________________

**Participant code _________**

Having been invited to participate in the study**:***“* **__________________________________”** I_____________________________________________ declare that:

1. I have been satisfactorily informed that the purpose of this research is to evaluate the implementation of the Indian ink technique as a point-of-care laboratory screening technique for cryptococcosis in HIV-infected adult patients in southern Mozambique;
2. I have been duly informed of the nature of my participation in this research, and the risks and benefits arising from it;
3. I understand that I will not receive any material or monetary reward for participating in the study;
4. I have been duly informed of my right to withdraw from the study at any time without any prejudice.
5. I understand that the information regarding my participation will be confidential, and that the information I will share will be used to meet the objectives of this study and also for decision-making by the Ministry of Health regarding future diagnoses of this disease (cryptococcosis) in the hospital.
6. I understand that I will participate in a 25-minute interview with audio recording: ( ) yes ( ) no.
7. I also understand that if I have any questions I can ask them by contacting the principal investigator in this study at any time, by telephone number: +258 847301265.
8. Or if you have any questions about your rights as a participant in this research, or if you feel that you have not been treated appropriately, you can contact the National Committee for Bioethics for Health (CNBS) of the Ministry of Health by calling 824066350. , which is located on Av. Eduardo Mondlane/Salvador Allende - Maputo, 2nd floor.

| ____________________________________  Participant signature___________________  Name (in capital letters) of the participant | _________________  Date and Time |
| --- | --- |

_________________________________________________ _________________

Signature of the person who provided the explanation of consent Date and Time

| ____________________________________________  Name (in capital letters) of the person who provided the explanation of consent |  |
| --- | --- |

**Anexo I Versão 04, Outubro 2023**

**QUESTIONÁRIO**

(Clínicos e Técnicos de Laboratório)

**Implementação da técnica de tinta da China em urina para triagem laboratorial *point-of-care* da Criptococose em pacientes adultos infectados por HIV no sul de Moçambique.**

1. **INFORMAÇÃO DEMOGRÁFICA**

| **N^0^** | **Informação Demográfica** | **Resposta** |
| --- | --- | --- |
| 01 | Código do participante | ___ ___ ___/_______/_____/2023 |
| 02. | Unidade Sanitária: | 🞏HCM 🞏HGM 🞏HGJM 🞏 HPM 🞏HPX 🞏HCC |
| 03. | Idade | <30  ≥30  <40  ≥40 |
| 04. | Sexo | 🞏 Masculino 🞏Feminino |
| 05 | Nacionalidade | 🞏Moçambicana  🞏Estrageira |
| 06. | Qual é a sua principal área de trabalho ou unidade no hospital? (Por favor, marque UMA resposta) | 🞏 Medicina  🞏 Laboratório  🞏 Outra (por favor especifique:____________ |
| 07. | Qual é o seu nível de instrução? | 🞏 Médico internista  🞏 Residente em pós-graduação  🞏 Médico generalista  🞏 Enfermeiro Superior  🞏 Enfermeiro médio  🞏 Técnico de Laboratório  🞏 Outro (por favor especifique): ________ |
| 08. | Anos de prática | 🞏< 1 🞏 ≥1 🞏<5 🞏≥5 |

**Anexo II**  **Versão 04, Outubro 2023**

**QUESTIONÁRIO**

**Código ___ ___ ___/_______/_____/202___**

(RESPONSÁVEL DO LABORATÓRIO E TÉCNICOS**)**

**B/F**

**Implementação da técnica de tinta da China em urina para triagem laboratorial *point-of-care* da Criptococose em pacientes adultos infectados por HIV no sul de Moçambique.**

**ANÁLISE DA SITUAÇÃO ACTUAL**

| Código da US/LAB______ | Nível_____ | N^o^ de técnicos______ |
| --- | --- | --- |

1. **Com que frequência é efectuado o controlo de qualidade**

Sempre As vezes**** Nunca Não sei ****

1. **Indique o equipamento e material existente no seu laboratório**

Incubador  Cabine de biossegurança  Centrifuga  Microscópio  Lâminas microscópicas  Lamelas  Geleira 

1. **Qual é a condição da infraestrutura?**

Adequado  Inadequado 

1. **Com que frequência o laboratório tem material/equipamento para teste de criptococose?**

Sempre As vezes**** Nunca Não sei ****

1. **Com que frequência o laboratório tem reagentes para teste de criptococose?**

Sempre As vezes**** Nunca Não sei ****

1. **Estima o número de amostras para o diagnóstico da criptococose que são processadas por semana no seu laboratório**

<10 10 -- 20 ≥30

1. **Com que frequência em amostras suspeitos, é diagnosticado *Criptococcus* spp no seu laboratório?**

 100%  80 – 99% 50 – 79%  <50%

1. **Considera a criptococose uma doença negligenciada em Moçambique?**

Sim Não  Não sei 

1. **Seu laboratório sofre atrasos na emissão resultados ou interrompe o processamento das amostras devido à falta de reagentes?**

Sim  Não  Não sei 

1. **Você sente que a qualidade do trabalho é consistente em todos os turnos? (horário de trabalho, fim de semana, feriado, plantão noturno)**

Sim Não

1. **Se NÃO, na sua opinião em qual turno a qualidade do serviço do laboratório é comprometida?**

Horário de trabalho Fim de semana Feriado Plantão noturno

1. **Com que frequência você recebe treinamento em microscopia?**

1 treino/ano 2 treinos/ano nunca fui treinado

1. **Quando foi o seu último treino?**

>6 meses  ≥1 ano  ≥2 anos

1. **Indique por favor o seu nível de instrução**

Médico especialista  Médico generalista  Enfermeiro Técnico de Laboratório

1. **No laboratório existe base de dados eletrónico e funcional?**

Sim  Não  Não sei 

1. **Existe no laboratório livro de registo de dados?**

Sim  Não  Não sei ****

1. **O laboratório tem critérios para rejeição de amostras**

Sim  Não **** Não sei ****

1. **Que tipo de amostras são processadas no laboratório?**

Sangue Urina LCR Expetoração Biopsia

Outras (por favor especifique)_____________________________

1. **Com que frequência tem o EPI disponível (luvas) para manuseamento das amostras?**

Sempre As vezes Nunca Não sei 

1. **Que tipo de exames estão disponíveis no laboratório para diagnóstico de criptococose**

Tinta da China  CrAg  Cultura  Teste de aglutinação em latex (TAL) Ensaio imune enzimático (EIE) PCR

1. **Que microrganismos de origem fúngica são pesquisados no Laboratório**

*Criptococcus spp Candida spp Aspergillus spp Pneumocistes jirovecii*  Outros (por favor especifique)____________________

1. **O laboratório tem procedimentos operacionais padrão (POPs) de diagnóstico?**

Sim Não

1. **Qual é o tempo de resposta do Laboratório** (forneça sua melhor estimativa)

____min _____ horas

1. **Com que frequência são discutidos internamente os resultados dos pacientes antes de partilha com os clínicos?**

Sempre As vezes Nunca Não sei 

1. **Indique a forma de partilha de resultados com clínicos (**pode escolher mais do que uma resposta)

WhatsApp Email  Plataforma (*website*)  Agente de serviço faz chegar ao clínico Outra (por favor especifique)___________________

1. **Seu laboratório autoclava/incinera amostras, culturas ou material infecioso antes de descartar?**

Sim Não Não sei

1. **Fornecimento de água corrente no seu laboratório**

<12h/dia 12 - 16h/dia 24h/dia

1. **Fornecimento de energia no seu laboratório**

<12h/dia 12 - 16h/dia 24h/dia

1. **Tem alternativa de fornecimento de energia (gerador)?**

Sim Não

1. **Que outros desafios/dificuldades você encontra para efectuar um exame de *Cryptococcus?***

**Obrigado pela colaboração.**

**Anexo III**  **Versão 04, Outubro 2023**

**GUIÃO DE ENTREVISTA AO CLÍNICO**

**Código ___ ___ ___/_______/_____/202___**

**Implementação da técnica de tinta da China em urina para triagem laboratorial *point-of-care* da Criptococose em pacientes adultos infectados por HIV no sul de Moçambique.**

**DIAGNÓSTICO CLÍNICO-LABORATORIAL E *FEEDBACK* DO LAB**

1. Encontra muitos casos de criptococose enquanto cuidas de pacientes no seu consultório?
2. Você se sente habilitado o suficiente para cuidar de pacientes com doença criptocócica?
3. Você se sente a vontade para cuidar de pacientes com doença criptocócica?
4. Que sinais e sintomas seus pacientes com doença criptocócica costumam apresentar?
5. Quais doenças imitam ou se parecem com a doença criptocócica no seu consultório?
6. Que tipo de teste habitualmente solicita quando suspeita uma doença criptocócica?
7. Na sua opinião o pessoal do laboratório está disponível quando você tenta contatá-los?
8. A punção lombar está sempre disponível na sua US?
9. Que desafios você encontra se tiver que referir pacientes para punção lombar com o outro médico?
10. Em que se apoia para o diagnóstico da criptococose na sua Unidade Sanitária?
11. Tem alguns critérios para decidir por um tratamento seja sindrómico em vez do direcionado?
12. Considera complexo ou simples o fluxo de atendimento incluindo diagnóstico laboratorial e tratamento?
13. O que constitui barreira no fluxo de atendimento, diagnóstico laboratorial e tratamento de pacientes suspeito da criptococose?
14. Na sua opinião, que aspectos podem facilitar a aderência ao novo teste de diagnóstico laboratorial da criptococose na sua US/LAB
15. Tendo em conta as dificuldades que motivações encontras no âmbito de atendimento e tratamento de pacientes suspeitos de criptococose?
16. Em que frequência as suas decisões terapêuticas foram suportados por Laboratório?
17. Estima o tempo de resposta Laboratorial para disponibilidade do resultado.

**Obrigado pela colaboração.**

**Anexo IV.**  **Versão 03, Setembro 2023**

**QUESTIONÁRIO**

**Código ___ ___ ___/_______/_____/202___**

**Implementação da técnica de tinta da China em urina para triagem laboratorial point-of-care da Criptococose em pacientes adultos infectados por HIV no sul de Moçambique**

**(Práticas/conduta _Clínico)**

1. **Indique o principal método de diagnóstico de criptococoses em uso no seu laboratório**

Clinico ; Tinta da China ; CrAg ; Cultura ; Teste de aglutinação em latex (TAL) ; Ensaio imune enzimático (EIE); PCR

1. **Com que frequência solicitas o teste?**

Sempre As vezes**** Nunca Não sei ****

1. **Com que frequência o teste de *Criptococcus* esta disponível?**

Sempre As vezes**** Nunca Não sei ****

1. **Tempo de resposta do Laboratório** (forneça sua melhor estimativa)

______min _______horas

1. **Tempo de início de tratamento após o resultado positivo** (forneça sua melhor estimativa)

______horas ______dias

1. **Considera a criptococose uma doença negligenciada em Moçambique?**

Sim Não 

1. **Antes de solicitar o teste que factores considera? (**pode escolher mais do que uma resposta)

**** Gravidade da doença

 Carga do trabalho na clínica/laboratório

 Idade do Paciente

 Disponibilidade de serviços de laboratório

CD4<100cell/mm^3^

CD4<100cell/mm^3^

 Outro (Faz favor mencionar):____________________________________________

1. **Qual é a sua opinião sobre o formulário de solicitação dos enxames laboratoriais?**

Adequado  Inadequado 

1. **O laboratório notifica as enfermarias quando há introdução de um novo exame incluindo os seus parâmetros?**

Sim Não

1. **O laboratório notifica imediatamente os resultados críticos**

Sim Não

1. **Com que frequência o laboratório avisa a você quando há interrupção de um determinado exame?**

Sempre As vezes Nunca Não sei 

1. **Você sente que a qualidade do trabalho é consistente em todos os turnos? (horário de trabalho, fim de semana, feriado, plantão noturno)**

Sim Não

1. **Se NÃO, na sua opinião em qual turno a qualidade do serviço é comprometida?**

Horário de trabalho Fim de semana Feriado Plantão noturno

1. **Quantos pacientes assiste por semana? (forneça sua melhor estimativa)**

<10 10 - 20 ≥30

1. **Qual é o perfil do paciente que chega á US**

Crítico moderado

1. **Assinale os sinais/sintomas da criptococose que observas nos seus pacientes**

cefaleia febre rigidez da nuca convulsões acne

Outro (por favor mencione):__________________________

1. **Existem critérios para triagem?**

Sim  Não 

1. **Guião de definição de caso disponível**

Sim  Não 

1. **O rastreio de criptococose em pacientes HIV faz parte da rotina na sua US?**

Sim  Não 

1. **O que constitui barreira para o diagnóstico e tratamento de pacientes suspeito da criptococose? (**pode escolher mais do que uma resposta)

 Baixa auto-confiança motivado por sobrecarga do trabalho no Laboratório

 Rotura de reagentes

 Rotura do teste

 Dificuldades para obtenção da amostra

Outro (Faz favor mencionar):_____________________________________________

1. **Com que frequência você confia completamente os resultados do teste do paciente**

 100%  80 – 99% 50 – 79%  <50%

1. **Quais factores podem fazer com que você trate criptococose na presença de um resultado** **negativo do teste?**

 Forte suspeita clínica

 O resultado do teste pode ser indeterminado, incorrecto ou inconsistente

 Idade do paciente

 Preferência

 Outro (Faz favor mencionar):____________________________________________

1. **O pessoal do laboratório está disponível quando você tenta contatá-los?**

Sim  Não 

1. **Na sua opinião o laboratório disponibiliza a segunda via do resultado quando você solicita?**

Sim  Não **** Nem sempre

1. **Anfotericina B esta geralmente disponível na Unidade sanitária?**

Sim  Não ** **Não sei Nem sempre

1. **Os pacientes podem adquirir fluconazol gratuitamente na Unidade sanitária?**

Sim  Não ** **Não sei

1. **Há registo de seguimento dos pacientes com resultados positivos para Criptococose**

 Sim

Não, os pacientes não retornam depois da primeira visita

1. **No livro tem informações detalhado de contacto dos pacientes positivos para *Criptococcus*?**

Sim  Não **** Não sei 

1. **Se for solicitado para melhorar algo no diagnóstico e tratamento da criptococose que alterações sugeria?**

**Obrigado pela colaboração.**

**Anexo V**  **Versão 04, Outubro 2023**

**Implementação da técnica de tinta da China em urina para triagem laboratorial *point-of-care* da Criptococose em pacientes adultos infectados por HIV no sul de Moçambique.**

**QUESTIONÁRIO**

**Código ___ ___ ___/_______/_____/202___**

**AVALIAÇÃO DA INTERVENÇÃO**

**(Clinico)**

1. **As ferramentas disponibilizadas no treino são mais esclarecedores no âmbito de diagnóstico e tratamento da criptococose**

Concordo fortemente Concordo Neutro Descordo Descordo fortemente

1. **A intervenção é uma alternativa recomendável para triagem laboratorial da Criptococose**

Concordo fortemente Concordo Neutro Descordo Descordo fortemente

1. **Desde a sua implementação o teste da tinta-da-China a partir da urina esteve sempre disponível no Hospital**

Concordo fortemente Concordo Neutro Descordo Descordo fortemente

1. **Qualidade dos serviços melhorou com o novo diagnóstico laboratorial**

Concordo fortemente Concordo Neutro Descordo Descordo fortemente

1. **Pode-se estimar a percentagem de pacientes beneficiados pela intervenção em:**

 100%  80 – 99% 50 – 79%  <50%

1. **Avalie a sua satisfação em relação ao diagnostico laboratorial com a técnica da tinta da China usando a urina**

Muito satisfeito Satisfeito Neutro insatisfeito Muito insatisfeito

1. **Avalie a sua satisfação em relação a disponibilidade do pessoal do laboratório quando você tenta contatá-los?**

Muito satisfeito Satisfeito Neutro insatisfeito Muito insatisfeito

1. **Avalie a sua satisfação em relação ao tempo de resposta do laboratório**

Muito satisfeito Satisfeito Neutro insatisfeito Muito insatisfeito

1. **Avalie a sua satisfação em relação ao formulário de solicitação dos enxames laboratoriais**

Muito satisfeito Satisfeito Neutro insatisfeito Muito insatisfeito

1. **Avalie a sua satisfação em relação a clareza, legibilidade e integridade do resultado do laboratório relatório?**

Muito satisfeito Satisfeito Neutro insatisfeito Muito insatisfeito

1. **A qualidade do diagnóstico foi consistente em todos os turnos? (horário de trabalho, fim de semana, feriado, plantão noturno)**

Concordo fortemente Concordo Neutro Descordo Descordo fortemente

1. **Na sua opinião em qual turno, a qualidade do serviço do laboratório foi comprometida?**

Horário de trabalho Fim de semana Feriado Plantão noturno NA

1. **O número de pacientes internados devido a complicações associadas a meningite criptocócica reduziu com a introdução da nova abordagem do diagnóstico**

Concordo fortemente Concordo Neutro Descordo Descordo fortemente

1. **Com a introdução da nova abordagem do diagnóstico o número de óbitos reduziu**

Concordo fortemente Concordo Neutro Descordo Descordo fortemente

1. **A nova abordagem do diagnóstico contribuiu para que maior número de indivíduos vivendo com HIV tivessem oportunidade de testar para *Criptococcus***

Concordo fortemente Concordo Neutro Descordo Descordo fortemente

1. **O tratamento de pacientes com meningite criptocócica melhorou com implementação da nova abordagem para diagnóstico laboratorial**

Concordo fortemente Concordo Neutro Descordo Descordo fortemente

1. **Avalie na generalidade a sua satisfação em relação a intervenção no seu hospital**

Muito satisfeito Satisfeito Neutro insatisfeito Muito insatisfeito

**Obrigado pela colaboração.**

**Anexo V**  **Versão 04, Outubro 2023**

**Implementação da técnica de tinta da China em urina para triagem laboratorial *point-of-care* da Criptococose em pacientes adultos infectados por HIV no sul de Moçambique.**

**QUESTIONÁRIO**

**Código ___ ___ ___/_______/_____/202___**

**AVALIAÇÃO DA INTERVENÇÃO**

**(Técnico de Laboratório)**

1. **Avalie o seu nível de confiança ao efectuar o exame de tinta de China usando urina**

****Muito confiante Confiante Neutro Inseguro Muito inseguro

1. **As ferramentas disponibilizadas no treino são mais esclarecedores no âmbito de diagnóstico e tratamento da criptococose**

Concordo fortemente Concordo Neutro Descordo Descordo fortemente

1. **A intervenção é uma alternativa recomendável para triagem laboratorial da Criptococose**

Concordo fortemente Concordo Neutro Descordo Descordo fortemente

1. **Desde a sua implementação o teste da tinta-da-China a partir da urina esteve sempre disponível no Hospital**

Concordo fortemente Concordo Neutro Descordo Descordo fortemente

1. **Qualidade dos serviços melhorou com o novo diagnóstico laboratorial**

Concordo fortemente Concordo Neutro Descordo Descordo fortemente

1. **Pode-se estimar a percentagem de pacientes beneficiados pela intervenção em:**

 100%  80 – 99% 50 – 79%  <50%

1. **Avalie a sua satisfação em relação ao diagnostico laboratorial com a técnica da tinta da China usando a urina**

Muito satisfeito Satisfeito Neutro insatisfeito Muito insatisfeito

1. **Avalie a sua satisfação em relação a clareza e legibilidade dos exames solicitados pelo clínico?**

Muito satisfeito Satisfeito Neutro insatisfeito Muito insatisfeito

1. **Avalie a sua satisfação em relação a disponibilidade dos clínicos quando você tenta contatá-los?**

Muito satisfeito Satisfeito Neutro insatisfeito Muito insatisfeito

1. **Avalie a sua satisfação em relação ao tempo de resposta do laboratório**

Muito satisfeito Satisfeito Neutro insatisfeito Muito insatisfeito

1. **Avalie a sua satisfação em relação ao formulário de solicitação dos enxames laboratoriais**

Muito satisfeito Satisfeito Neutro insatisfeito Muito insatisfeito

1. **A qualidade do diagnóstico foi consistente em todos os turnos? (horário de trabalho, fim de semana, feriado, plantão noturno)**

Concordo fortemente Concordo Neutro Descordo Descordo fortemente

1. **Na sua opinião em qual turno, a qualidade do serviço do laboratório foi comprometida?**

Horário de trabalho Fim de semana Feriado Plantão noturno NA

1. **Com a introdução da nova abordagem do diagnóstico o número de óbitos reduziu**

Concordo fortemente Concordo Neutro Descordo Descordo fortemente

1. **A nova abordagem do diagnóstico contribuiu para que maior número de indivíduos vivendo com HIV tivessem oportunidade de testar para *Criptococcus***

Concordo fortemente Concordo Neutro Descordo Descordo fortemente

1. **Avalie na generalidade a sua satisfação em relação a intervenção no seu hospital**

Muito satisfeito Satisfeito Neutro insatisfeito Muito insatisfeito

**Obrigado pela colaboração.**
